# Supplementary figures and images for: Epimutations driven by RNAi or heterochromatin evoke transient antimicrobial drug resistance in pathogenic Mucor fungi
Source: PLoS Biol. 2026 Feb 2;24(2):e3003598. doi: 10.1371/journal.pbio.3003598 (PMC12863538; doi:10.1371/journal.pbio.3003598)

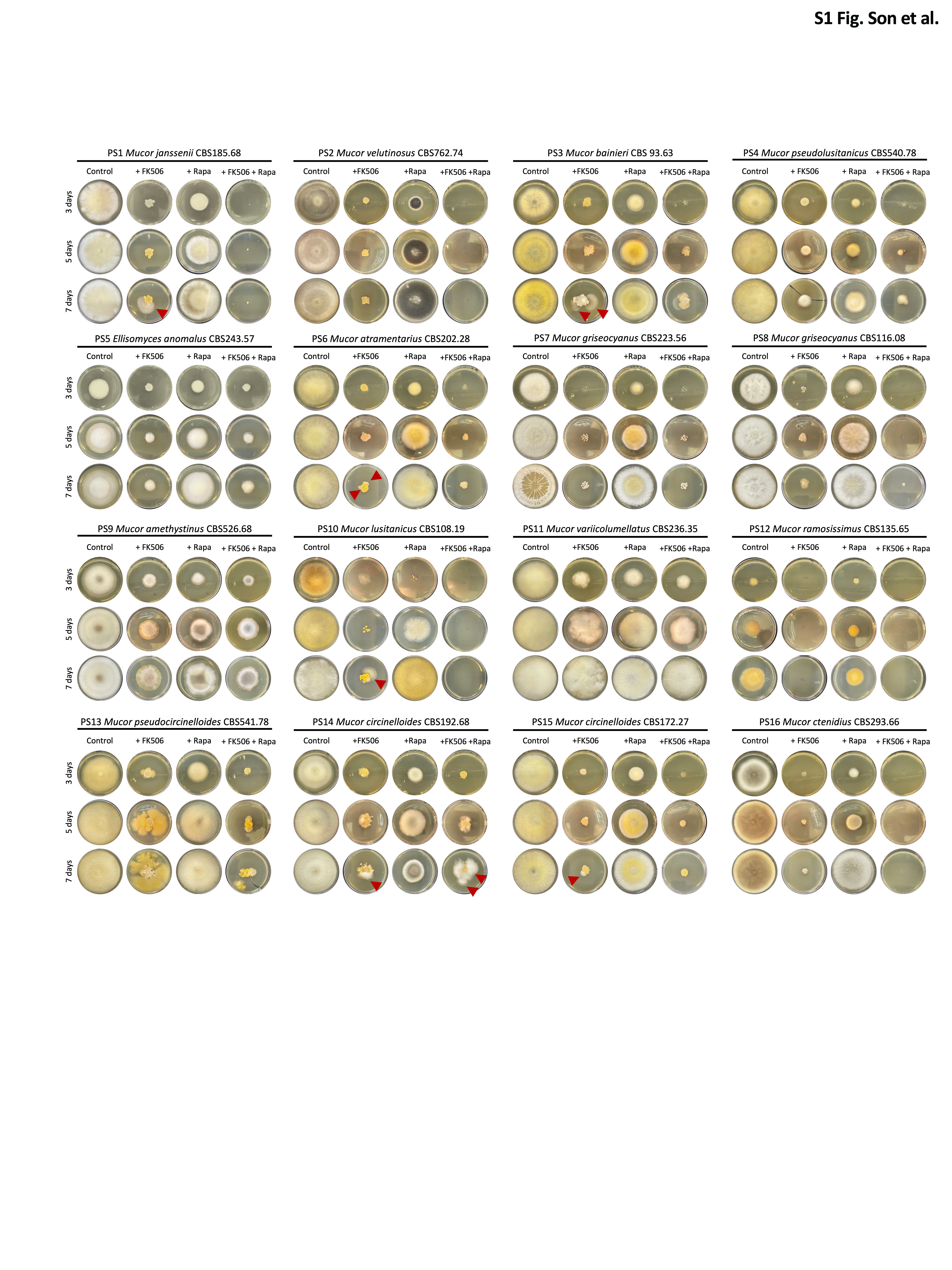

Supplement: S1 Fig — Spores from phylogenetic species (PS) 1 to PS16 were point-inoculated onto YPD plates and incubated at room temperature until resistant colonies emerged. After seven days, resistant colonies were observed in PS1, PS3, PS6, PS10, PS13, PS14, and PS15. PS10, PS14, and PS15 were included as positive controls, as resistant strains had previously been characterized. Red arrows indicate sectors of FK506-resistant growth on the plate. FK506 was at a concentration of 1 μg/mL, while rapamycin was used at a concentration of 100 ng/mL. (TIFF) [file pbio.3003598.s001.tiff]

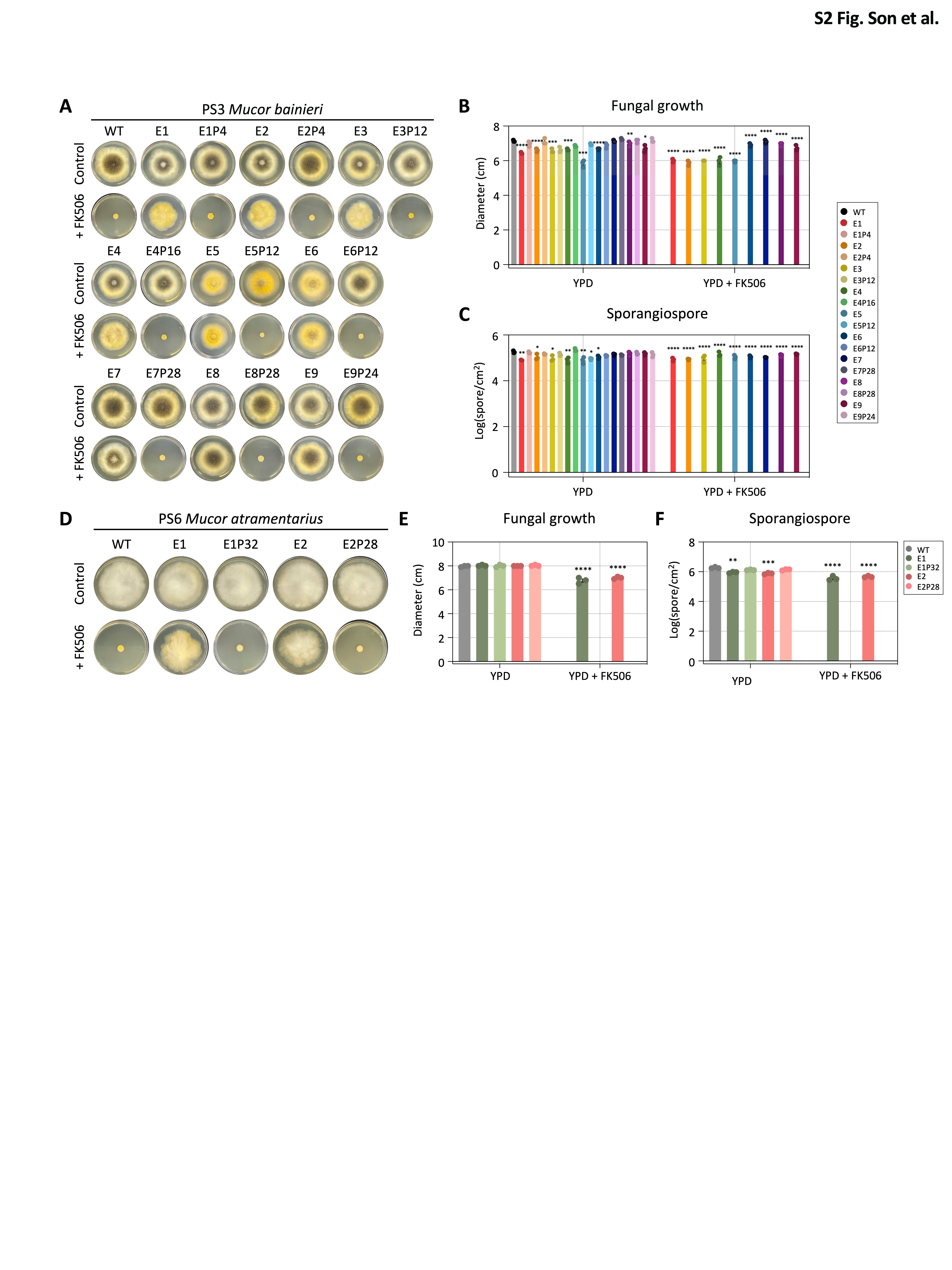

Supplement: S2 Fig — (A, D) Point-inoculation phenotypes of Mucor strains inoculated with 106 spores and grown on YPD with or without 1 μg/mL FK506 for 4 days. (B, E) Quantification of fungal growth for the same strains shown in (A or D). Error bars represent mean ± SEM (n = 3). Statistical significance: *p ≤ 0.05; **p ≤ 0.01; ***p ≤ 0.001; ****p ≤ 0.0001. (C, F) Quantification of sporangiospore production for the same strains shown in (A or D). Error bars represent mean ± SEM (n = 3). Statistical significance: *p ≤ 0.05; **p ≤ 0.01; ***p ≤ 0.001; ****p ≤ 0.0001. WT, wild-type; E1–E9, epimutants; E1P4–E9P24, revertants. The data underlying this figure can be found in S1 Data. (TIFF) [file pbio.3003598.s002.tiff]

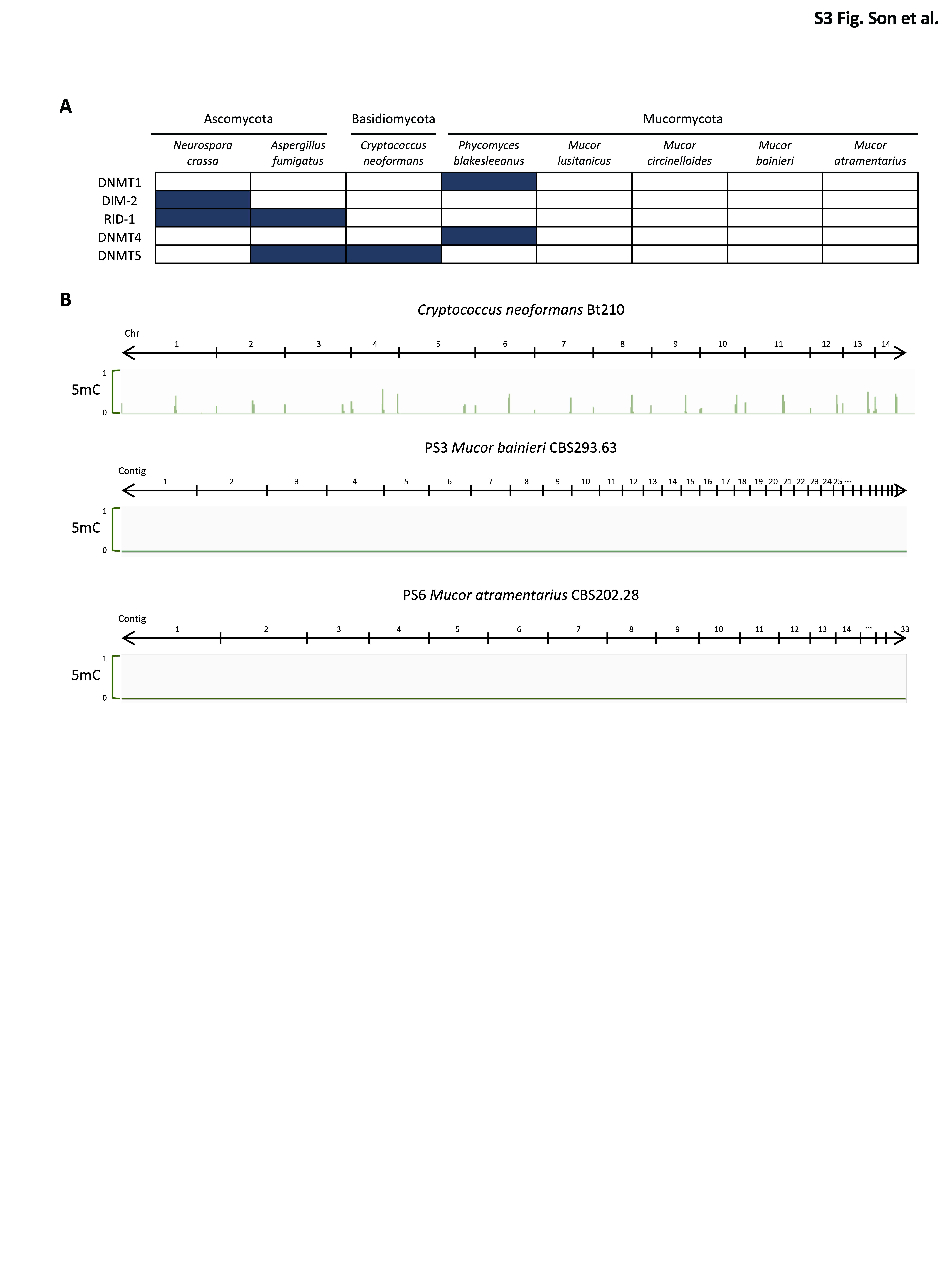

Supplement: S3 Fig — (A) Presence/absence matrix of 5mC DNA methyltransferases across multiple fungal species. Dark blue indicates the presence of an ortholog, whereas light blue indicates its absence. (B) Genome-wide distribution of 5mC in wild-type strain of Cryptococcus neoformans Bt210 (positive control), M. bainieri, and M. atramentarius, detected from Oxford Nanopore sequencing. 5mC was identified using the Dorado basecaller with modified-base models and summarized with modkit pileup under default settings. Nanopore sequencing data of C. neoformans Bt210 (published in Huang J and colleagues, PNAS, 2024; PMID: 39536081; NCBI BioProject PRJNA1138746) were used as a positive control for the analysis of 5mC in two Mucor species. (TIFF) [file pbio.3003598.s003.tiff]

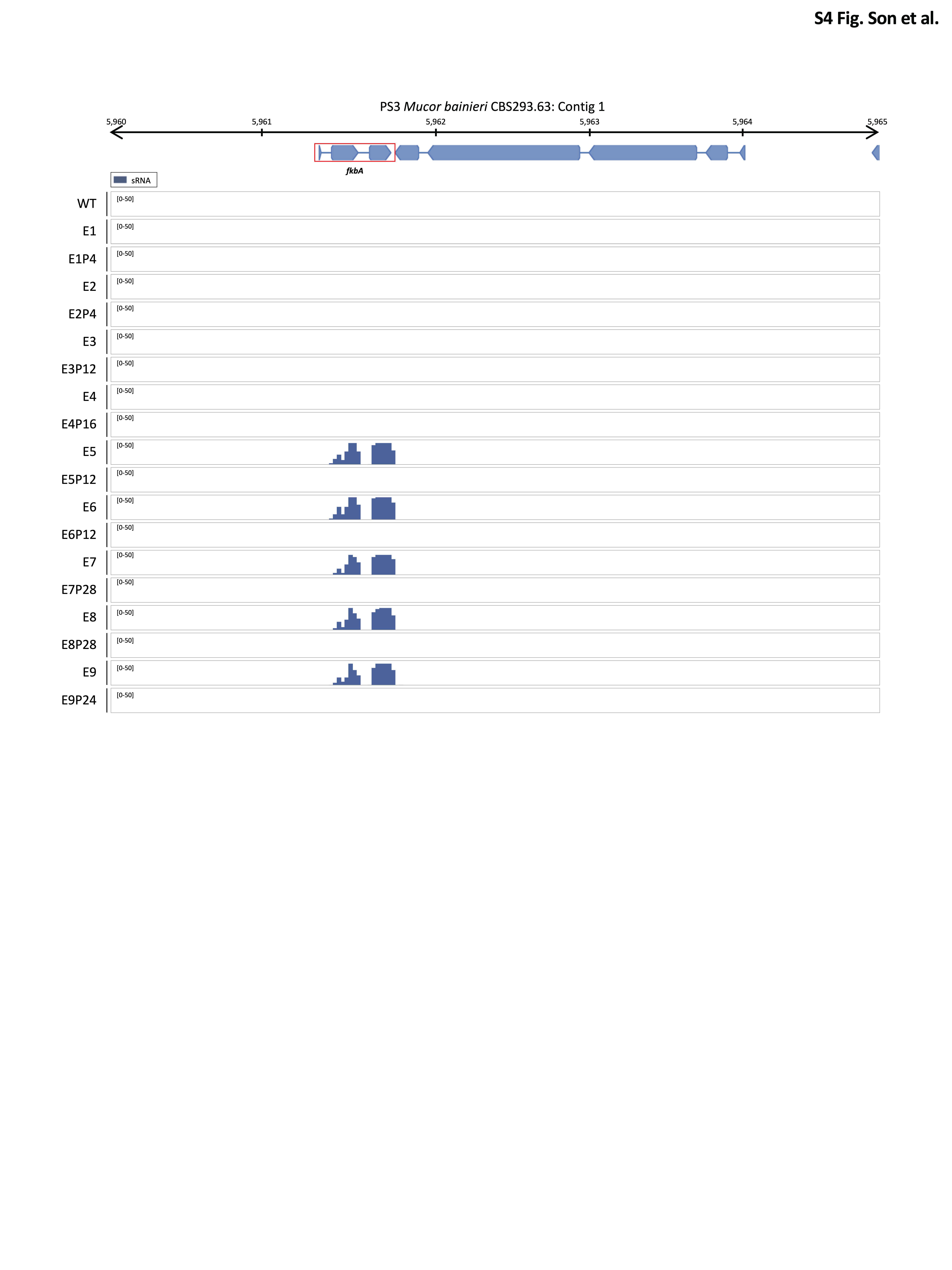

Supplement: S4 Fig — Small RNA coverage mapped across the fkbA gene and its neighboring loci. The genomic plot shows a 5 kb region encompassing the fkbA locus, including the fkbA gene (red box) and the adjacent patA gene (PS3_001330). (TIFF) [file pbio.3003598.s004.tiff]

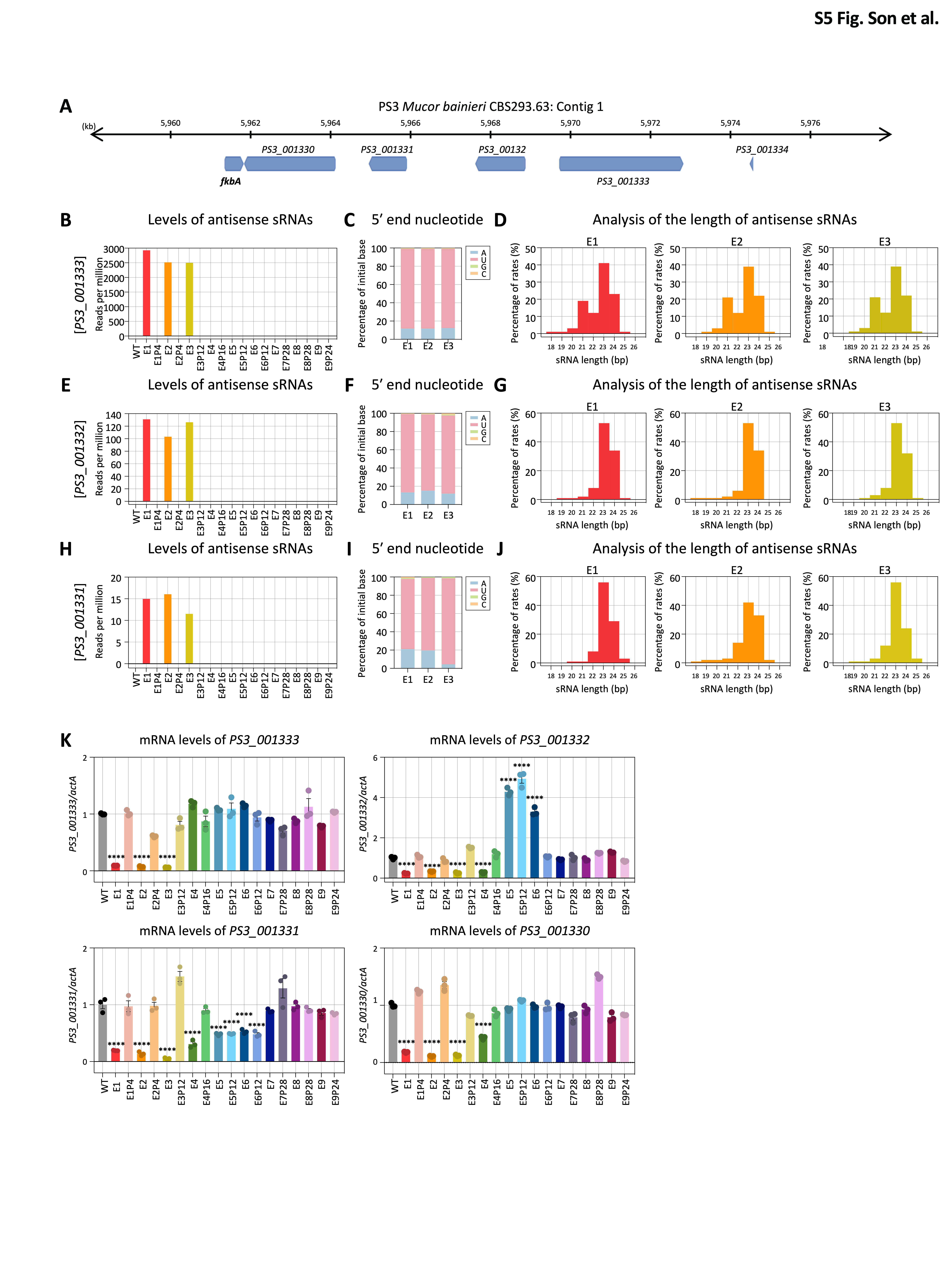

Supplement: S5 Fig — (A) Genomic schematic of the fkbA locus and its flanking genes. (B, E, H) Antisense sRNA abundance (RPM: reads per million) mapped to genes located upstream and downstream of the fkbA. (C, F, I) 5′ end nucleotide preference of antisense sRNAs associated with each fkbA-neighboring gene. (D, G, J) Size distribution of antisense sRNAs mapped to each fkbA-neighboring gene. (K) Quantification of mRNA expression in genes adjacent to the fkbA in WT, epimutants, and revertants. Error bars represent mean ± SEM (n = 3). Statistical significance: ****p ≤ 0.0001. WT, wild-type; E1–E9, epimutants; E1P4–E9P24, revertants. The data underlying this figure can be found in S1 Data. (TIFF) [file pbio.3003598.s005.tiff]

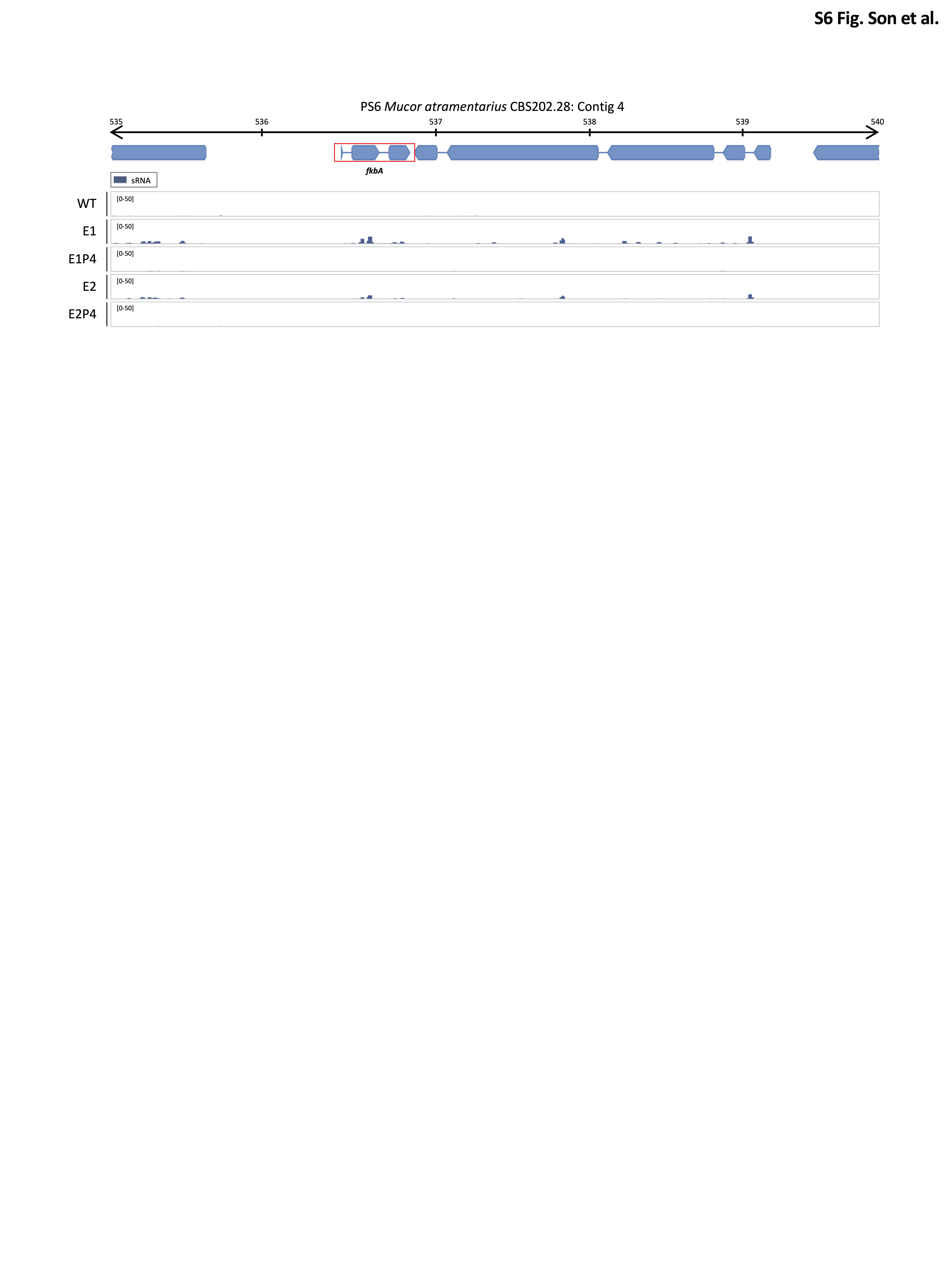

Supplement: S6 Fig — Small RNA coverage mapped across the fkbA gene and its neighboring loci. The genomic plot shows a 5 kb region encompassing the fkbA locus, including the fkbA gene (red box) and the adjacent patA gene (PS6_003788). (TIFF) [file pbio.3003598.s006.tiff]

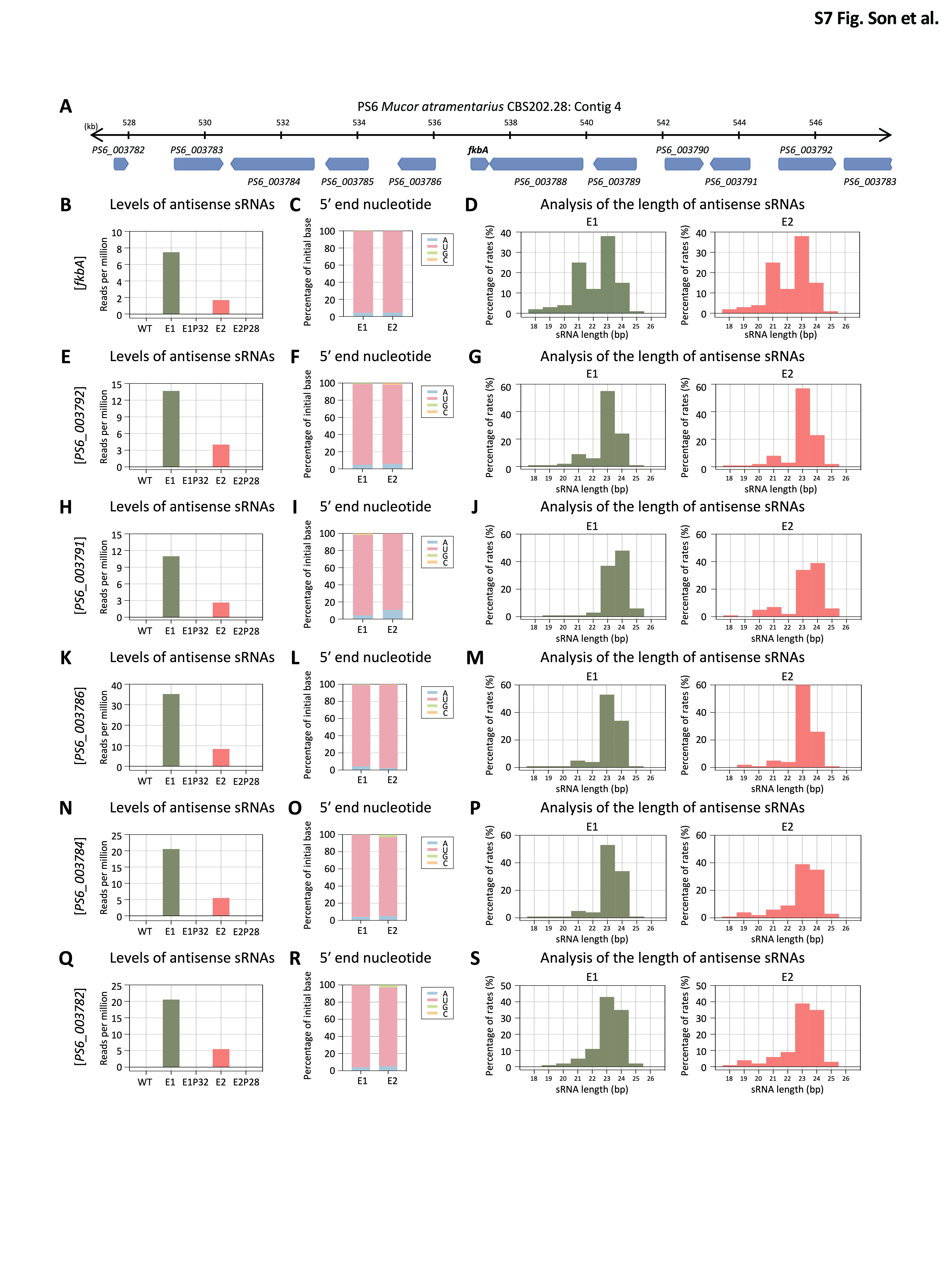

Supplement: S7 Fig — (A) Genomic schematic of the fkbA locus and its flanking genes. (B, E, H, K, N, Q) Antisense sRNA abundance (RPM: reads per million) mapped to the fkbA gene and its upstream and downstream neighboring genes. (C, F, I, L, O, R) 5′ end nucleotide preference of antisense sRNAs associated with the fkbA gene and each fkbA-neighboring gene. (D, G, J, M, P, S) Size distribution of antisense sRNAs mapped to the fkbA gene and each fkbA-neighboring gene. WT, wild-type; E1 and E2, epimutants; E1P32 and E2P28, revertants. The data underlying this figure can be found in S1 Data. (TIFF) [file pbio.3003598.s007.tiff]

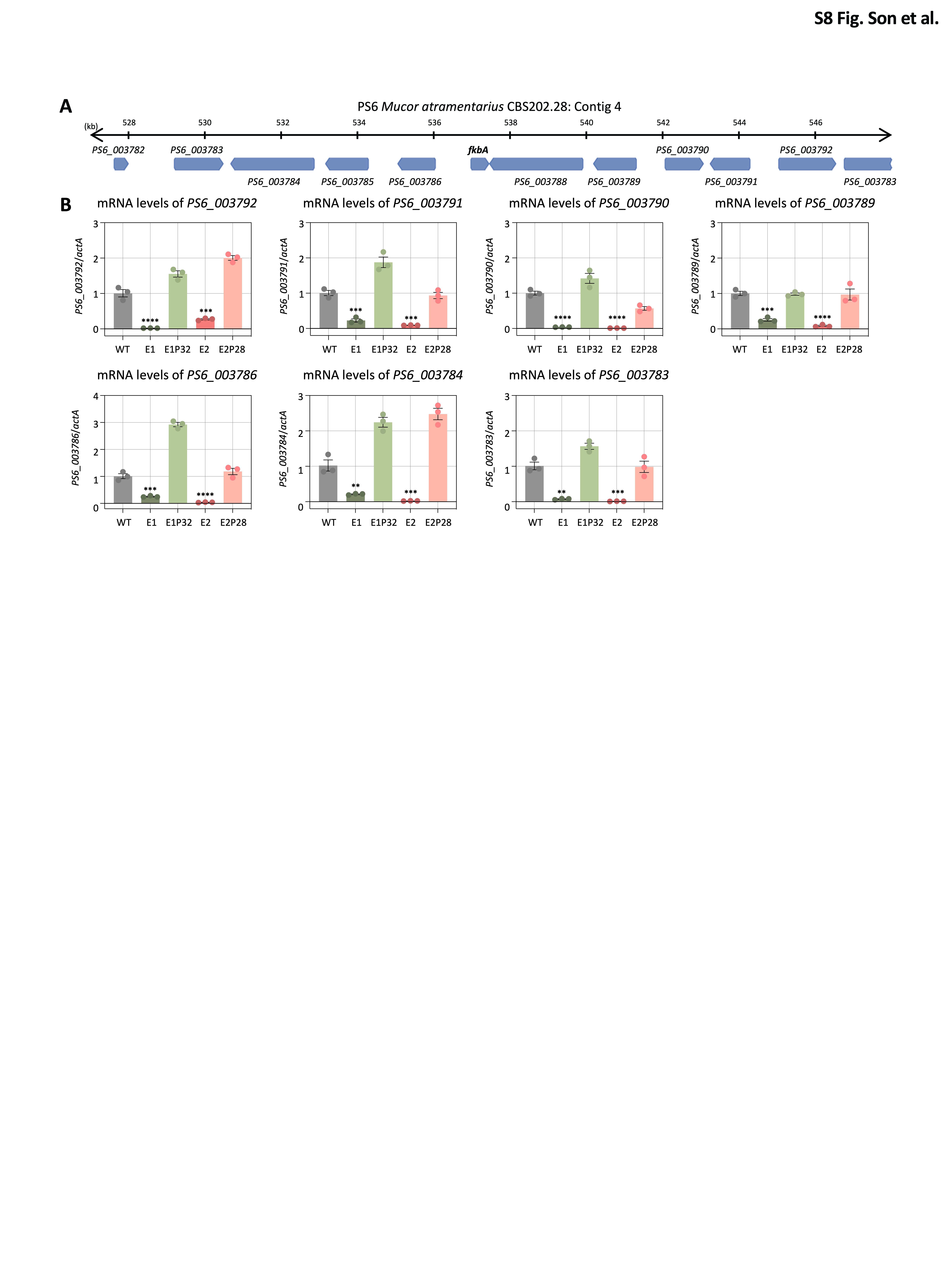

Supplement: S8 Fig — (A) Genomic schematic of the fkbA locus and its flanking genes. (B) Quantification of mRNA expression in genes adjacent to the fkbA in WT, epimutants, and revertants. Error bars represent mean ± SEM (n = 3). Statistical significance: **p ≤ 0.01, ***p ≤ 0.001, and ****p ≤ 0.0001. WT, wild-type; E1 and E2, epimutants; E1P32 and E2P28, revertants. The data underlying this figure can be found in S1 Data. (TIFF) [file pbio.3003598.s008.tiff]

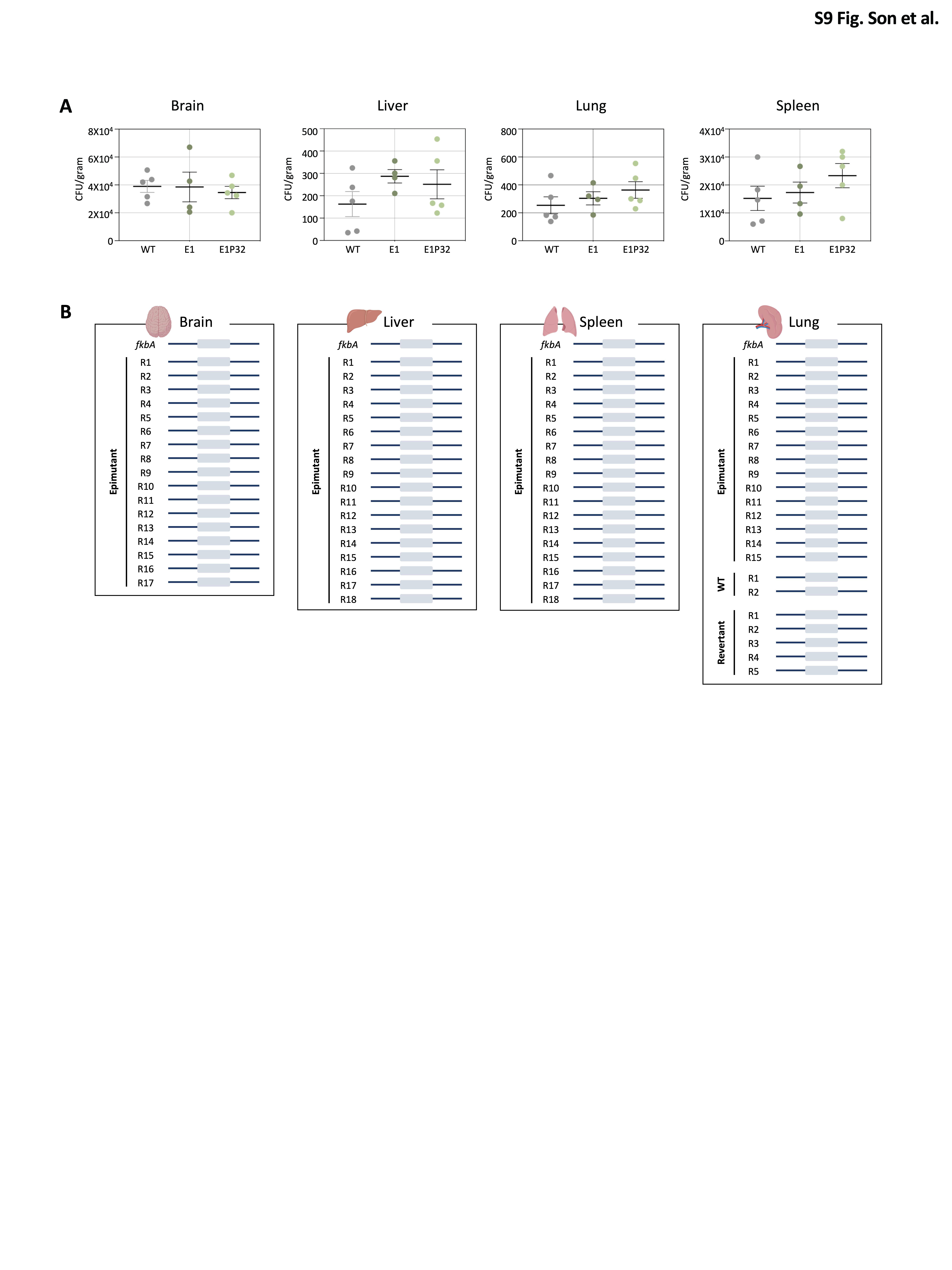

Supplement: S9 Fig — (A) Fungal burden following retro-orbital inoculation of M. atramentarius with cyclophosphamide-treated mice (200 mg/kg) was determined at 4 days post-infection. Fungal burden was expressed as colony-forming units (CFU) per gram of tissue across four organs. Five biological replicates were assessed. Error bars represent mean ± SEM (n = 5). WT, wild-type; E1, epimutant; E1P32, revertant. (B) Schematic representation of Nanopore sequencing results showing the presence or absence of point mutations across the fkbA locus in FK506-resistant colonies recovered from each organ after in vivo infection. PCR amplicons spanning the promoter, open reading frame, and terminator regions of the fkbA locus were subjected to long-read Nanopore sequencing, and individual reads were mapped to the reference genome to assess nucleotide variation across the locus. No mutations in the fkbA locus were detected. Organ diagrams were generated with BioRender.com. The data underlying this figure can be found in S1 Data. (TIFF) [file pbio.3003598.s009.tiff]

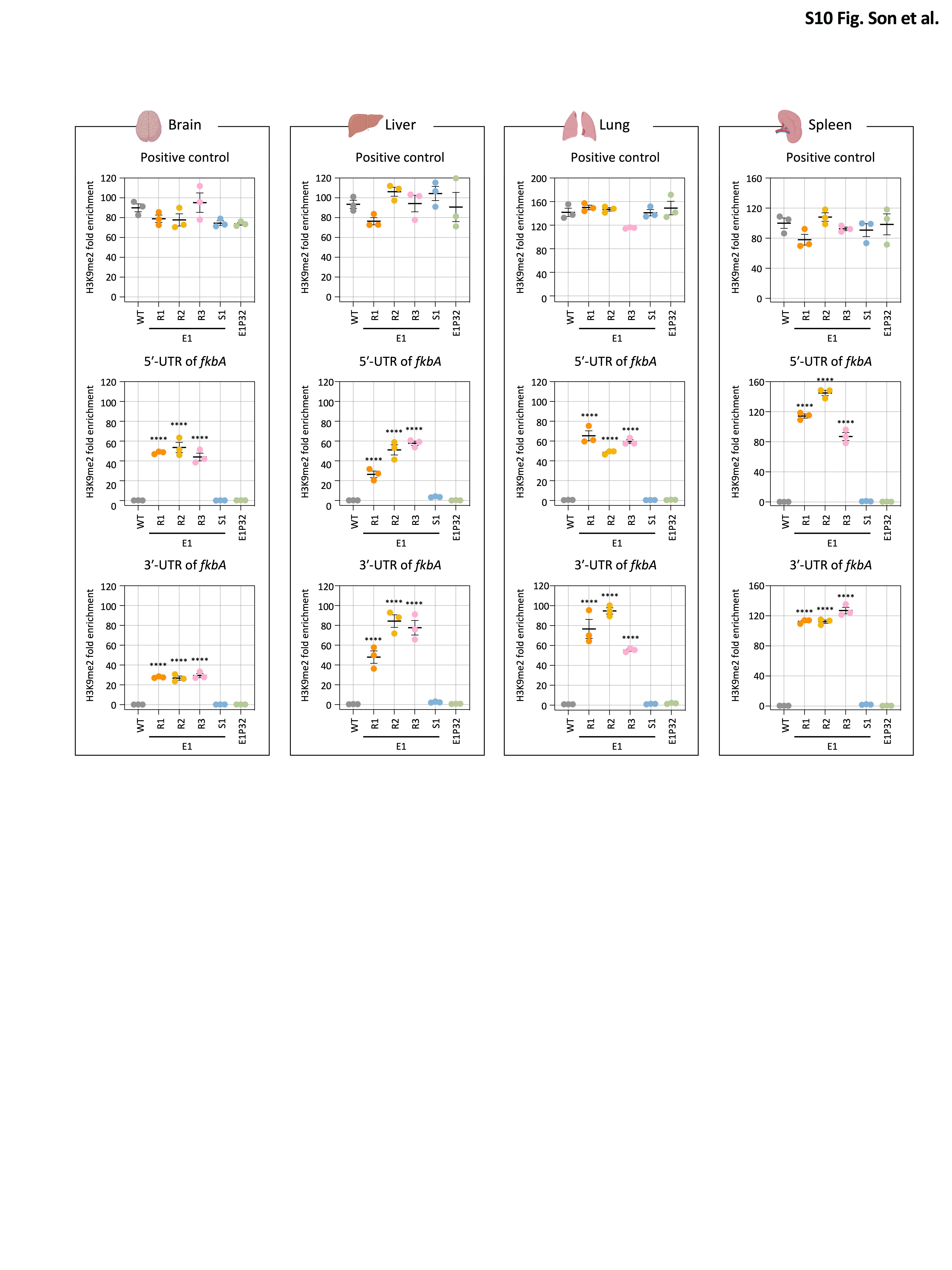

Supplement: S10 Fig — ChIP-qPCR analysis of H3K9me2 enrichment at the fkbA locus following in vivo passage, targeting the positive control region, 5′ UTR, and 3′ UTR. Error bars represent mean ± SEM (n = 3). Statistical significance: ***p ≤ 0.001; ****p ≤ 0.0001. WT, wild-type; E1R1–E1R3, FK506-resistant epimutants; E1S1, FK506-sensitive epimutant; E1P32, revertant. Organ diagrams were generated with BioRender.com. The data underlying this figure can be found in S1 Data. (TIFF) [file pbio.3003598.s010.tiff]

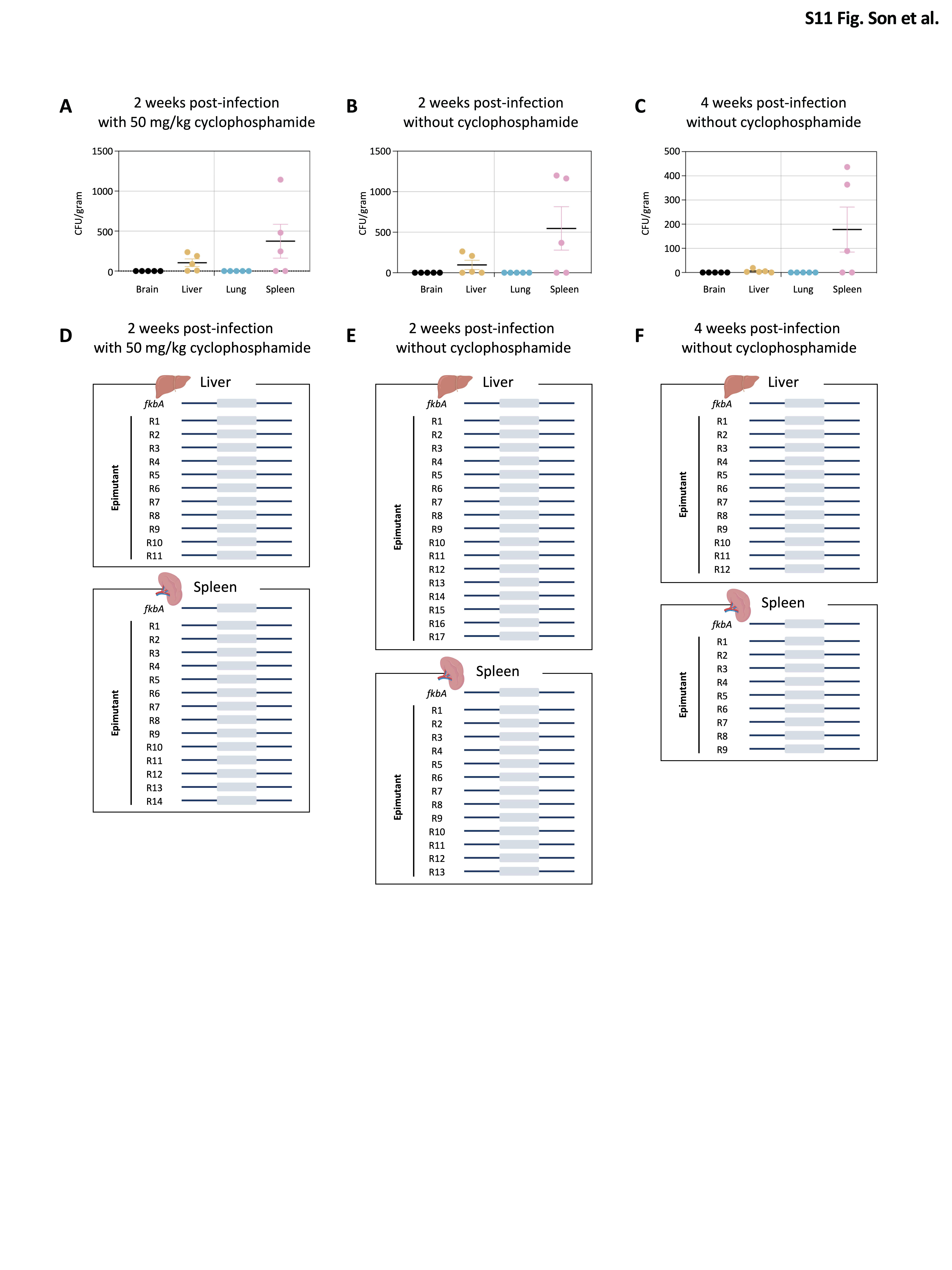

Supplement: S11 Fig — (A) Fungal burden following retro-orbital inoculation of M. atramentarius epimutant in cyclophosphamide-treated mice (50 mg/kg) at 2 weeks post-infection. (B) Fungal burden in untreated mice at 2 weeks post-infection. (C) Fungal burden in untreated mice at 4 weeks post-infection. For all panels, fungal burdens were expressed as colony-forming units (CFU) per gram of tissue across four organs. Five biological replicates were assessed. Error bars represent mean ± SEM (n = 5). (D–F) Schematic representation of Nanopore sequencing results showing the presence or absence of point mutations across the fkbA locus in FK506-resistant colonies recovered from each organ after in vivo infection. PCR amplicons spanning the promoter, open reading frame, and terminator regions of the fkbA locus were subjected to long-read Nanopore sequencing, and individual reads were mapped to the reference genome to assess nucleotide variation across the locus. No mutations in the fkbA locus were detected. Organ diagrams were generated with BioRender.com. The data underlying this figure can be found in S1 Data. (TIFF) [file pbio.3003598.s011.tiff]

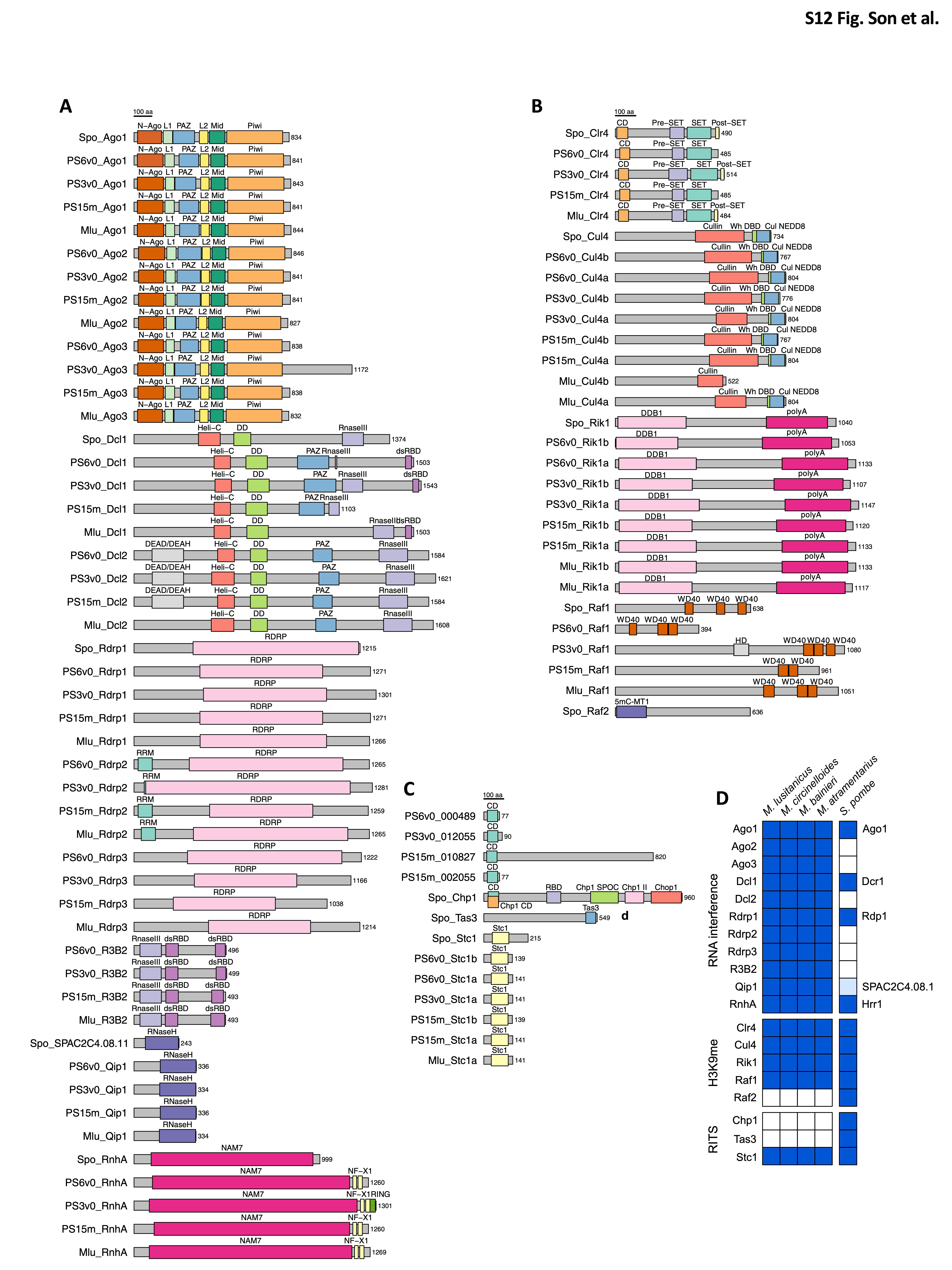

Supplement: S12 Fig — (A–C) Proteins that mediate (A) RNA interference, (B) H3K9 methylation-based heterochromatin formation, and (C) RNA-induced transcriptional silencing (RITS) are depicted. Rectangles display the full-length, scaled protein sequences of every identified protein homolog and their predicted, color-coded InterPro protein domains abbreviated as follows: N-Ago (Protein argonaute, N-terminal, IPR032474); L1 (Argonaute, linker 1 domain, IPR014811); PAZ (PAZ domain, IPR003100); L2 (Argonaute linker 2 domain, IPR032472); Mid (Protein argonaute, Mid domain, IPR032473); Piwi (Piwi domain, IPR003165); DEAD/DEAH (DEAD/DEAH box helicase domain, IPR011545); Heli_C (Helicase C-terminal domain-like, IPR001650); DD (Dicer dimerization domain, IPR005034); RNaseIII (Ribonuclease III domain, IPR000999); RRM (RNA recognition motif domain, IPR000504); RDRP (RNA-dependent RNA polymerase, eukaryotic type, IPR007855); RNaseH (Ribonuclease H superfamily, IPR036397); NAM7 (DNA2/NAM7-like helicase, IPR045055); NF-X1 (Zinc finger, NF-X1-type, IPR000967); dsRBD (Double-stranded RNA-binding domain, IPR014720); RING (Zinc finger, RING-type, IPR001841); SET (SET domain, IPR001214); Pre-SET (Pre-SET domain, IPR007728); Post-SET (Post-SET domain, IPR003616); Cul_NEDD8 (Cullin protein, neddylation domain, IPR019559); Cullin (Cullin homology domain, IPR016158); Wh DBD (Winged helix DNA-binding domain superfamily, IPR036390); CD (Chromo domain, IPR023780); Stc1 (Stc1 domain, IPR024630); RBD (RNA-binding domain superfamily, IPR035979); Chop1 (Chop1, PIN domain, IPR048709); Tas3 (Tas3, C-terminal helical domain, IPR049112); Chp1 CD (Chp1, chromodomain, IPR049937); Chp1 SPOC (Chp1, SPOC domain, IPR049938); Chp1 II (Chp1, domain II, IPR056341). (D) A matrix displays the presence (blue) or absence (white) of the proteins described in (a–c) across M. lusitanicus PS10, M. circinelloides PS15, M. bainieri PS3, and M. atramentarius PS6, together with Schizosaccharomyces pombe as reference. A light shade of blue rep [file pbio.3003598.s012.tiff]

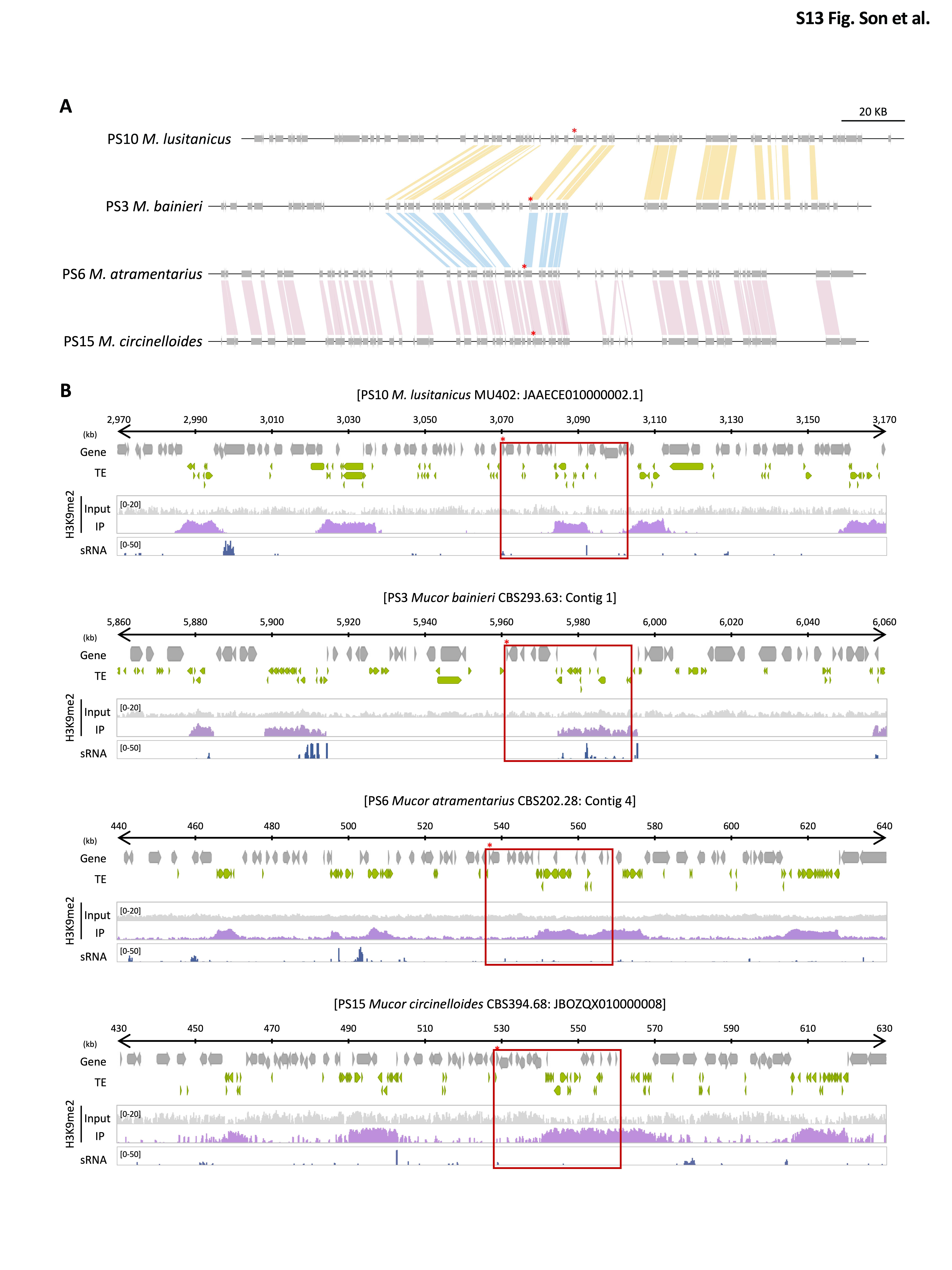

Supplement: S13 Fig — (A) Comparative synteny analysis of genomic regions flanking the fkbA locus in four Mucor species. Homologous genes were initially identified with DIAMOND protein alignment and validated with BLASTP, and synteny blocks were visualized in R. The red asterisk denotes the fkbA gene. (B) ChIP-seq profiles of H3K9me2 and small RNA coverage across the fkbA locus in the four Mucor species (M. bainieri, M. atramentarius, M. lusitanicus, and M. circinelloides). The red asterisk denotes the fkbA gene. Red boxes highlight a region adjacent to fkbA that contains a cluster of transposable elements enriched with H3K9me2. H3K9me2 ChIP-seq and small RNA sequencing data for M. lusitanicus and M. circinelloides were downloaded from NCBI BioProjects PRJNA903107 and PRJNA1170303, respectively, and analyzed with the same pipeline as applied for M. bainieri and M. atramentarius. (TIFF) [file pbio.3003598.s013.tiff]

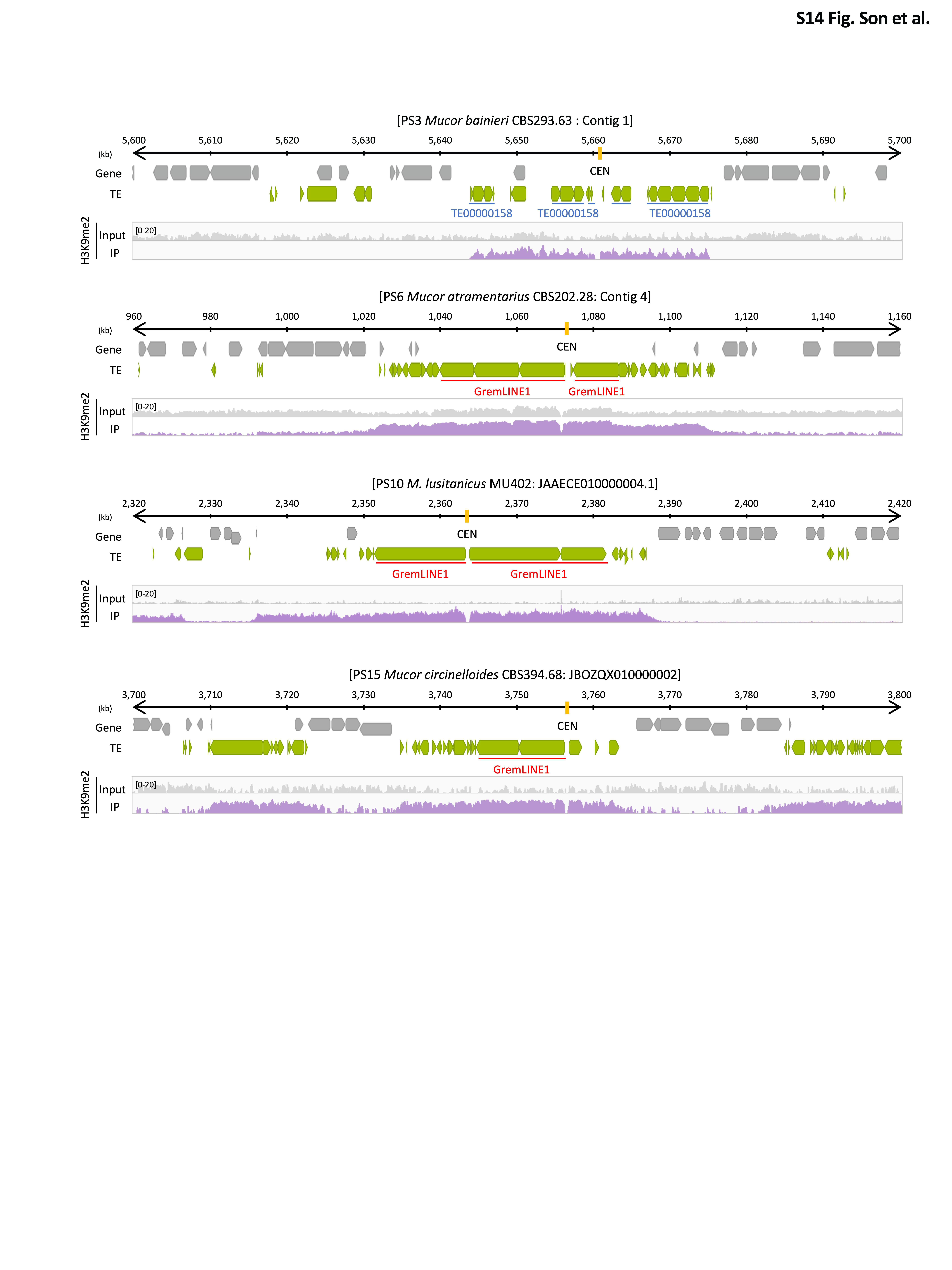

Supplement: S14 Fig — Genomic views display gene annotations, TE distribution, CEN boundaries, H3K9me2 ChIP enrichment, and sRNA accumulation in the four Mucor species (M. bainieri, M. atramentarius, M. lusitanicus, and M. circinelloides). GremLINE1 elements, highlighted in red, are present within or near putative centromeres in three Mucor species (M. atramentarius, M. lusitanicus, and M. circinelloides), but are absent in the M. bainieri genome. Instead, the putative centromeric region of M. bainieri is enriched for a distinct repeat fragment, TE00000158. Putative centromere regions were predicted based on the M. lusitanicus CEN motif, reported in Navarro-Mendoza and colleagues, Curr Biol., 2019 (PMID: 31679929). (TIFF) [file pbio.3003598.s014.tiff]
