# Supplementary material for: Epimutations driven by RNAi or heterochromatin evoke transient antimicrobial drug resistance in pathogenic Mucor fungi
Source: PLoS Biol. 2026 Feb 2;24(2):e3003598. doi: 10.1371/journal.pbio.3003598 (PMC12863538; doi:10.1371/journal.pbio.3003598)
Supplement: S1 Table — (DOCX) [file pbio.3003598.s015.docx]

**S1 Table. *Mucor* strains analyzed in this study.**

| Species | Strain name | Description | References |
| --- | --- | --- | --- |
| *Mucor janssenii* | CBS 185.68 | Phylogenetic species 1 | ^1^ |
|  | YES1 | Mutant 1 (M1) | This study |
|  | YES2 | Mutant 2 (M2) | This study |
|  | YES3 | Mutant 3 (M3) | This study |
|  | YES4 | Mutant 4 (M4) | This study |
|  | YES5 | Mutant 5 (M5) | This study |
|  | YES6 | Mutant 6 (M6) | This study |
|  | YES7 | Mutant 7 (M7) | This study |
|  | YES8 | Mutant 8 (M8) | This study |
|  | YES9 | Mutant 9 (M9) | This study |
|  | YES10 | Mutant 10 (M10) | This study |
| *Mucor velutinosus* | CBS 762.74 | Phylogenetic species 2 | ^1^ |
| *Mucor bainieri* | CBS 293.63 | Phylogenetic species 3 | ^1^ |
|  | YES12 | Epimutant 1 (E1) | This study |
|  | YES13 | Epimutant 2 (E2) | This study |
|  | YES14 | Epimutant 3 (E3) | This study |
|  | YES15 | Epimutant 4 (E4) | This study |
|  | YES16 | Epimutant 5 (E5) | This study |
|  | YES17 | Epimutant 6 (E6) | This study |
|  | YES18 | Mutant 1 (M1) | This study |
|  | YES19 | Epimutant 7 (E7) | This study |
|  | YES20 | Epimutant 8 (E8) | This study |
|  | YES21 | Epimutant 9 (E9) | This study |
|  | YES22 | Revertant after passage 4 of Epimutant 1 | This study |
|  | YES23 | Revertant after passage 4 of Epimutant 2 | This study |
|  | YES24 | Revertant after passage 12 of Epimutant 3 | This study |
|  | YES25 | Revertant after passage 16 of Epimutant 4 | This study |
|  | YES26 | Revertant after passage 12 of Epimutant 5 | This study |
|  | YES27 | Revertant after passage 12 of Epimutant 6 | This study |
|  | YES28 | Revertant after passage 28 of Epimutant 7 | This study |
|  | YES29 | Revertant after passage 28 of Epimutant 8 | This study |
|  | YES30 | Revertant after passage 24 of Epimutant 9 | This study |
| *Mucor pseudolusitanicus* | CBS 540.78 | Phylogenetic species 4 | ^1^ |
| *Ellisomyces anomalus* | CBS 243.57 | Phylogenetic species 5 | ^1^ |
| *Mucor atramentarius* | CBS 202.28 | Phylogenetic species 6 | ^1^ |
|  | YES31 | Mutant 1 (M1) | This study |
|  | YES32 | Mutant 2 (M2) | This study |
|  | YES33 | Mutant 3 (M3) | This study |
|  | YES34 | Mutant 4 (M4) | This study |
|  | YES35 | Mutant 5 (M5) | This study |
|  | YES36 | Mutant 6 (M6) | This study |
|  | YES37 | Mutant 7 (M7) | This study |
|  | YES38 | Epimutant 1 (E1) | This study |
|  | YES39 | Epimutant 2 (E2) | This study |
|  | YES40 | Mutant 8 (M8) | This study |
|  | YES41 | Revertant after passage 32 of Epimutant 1 | This study |
|  | YES42 | Revertant after passage 28 of Epimutant 2 | This study |
| *Mucor griseocyanus* | CBS 223.56 | Phylogenetic species 7 | ^1^ |
| *Mucor griseocyanus* | CBS 116.08 | Phylogenetic species 8 | ^1^ |
| *Mucor amethystinus* | CBS 526.68 | Phylogenetic species 9 | ^1^ |
| *Mucor lusitanicus* | CBS 108.19 | Phylogenetic species 10 | ^1^ |
| *Mucor variicolumellatus* | CBS 236.35 | Phylogenetic species 11 | ^1^ |
| *Mucor ramosissimus* | CBS 135.65 | Phylogenetic species 12 | ^1^ |
| *Mucor pseudocircinelloides* | CBS 541.78 | Phylogenetic species 13 | ^1^ |
| *Mucor circinelloides* | CBS 192.68 | Phylogenetic species 14 | ^1^ |
| *Mucor circinelloides* | CBS 172.27 | Phylogenetic species 15 | ^1^ |
| *Mucor circinelloides* | CBS 394.68 | Phylogenetic species 15 | ^1^ |
| *Mucor ctenidius* | CBS 293.66 | Phylogenetic species 16 | ^1^ |

**Reference**

1. Wagner, L., Stielow, J.B., de Hoog, G.S., Bensch, K., Schwartze, V.U., Voigt, K., Alastruey-Izquierdo, A., Kurzai, O., and Walther, G. (2020). A new species concept for the clinically relevant *Mucor circinelloides* complex. Persoonia 44, 67-97. https://doi.org/10.3767/persoonia.2020.44.03.
