# Supplementary material for: Epimutations driven by RNAi or heterochromatin evoke transient antimicrobial drug resistance in pathogenic Mucor fungi
Source: PLoS Biol. 2026 Feb 2;24(2):e3003598. doi: 10.1371/journal.pbio.3003598 (PMC12863538; doi:10.1371/journal.pbio.3003598)
Supplement: S2 Table — (DOCX) [file pbio.3003598.s016.docx]

**S2 Table. Oligonucleotides utilized in this study.**

| **Oligo Name** | **Sequence 5' to 3'** | **Description** |
| --- | --- | --- |
| JOHE53988/YES87 | GGATCAGATGCTGCTTGCC | PS1 *fkbA* seq F |
| JOHE53989/YES88 | ATGGGCTTGTTGCATCGC | PS1 *fkbA* seq R |
| JOHE54708/YES228 | GCTCTTGATGCTGTGCTGTAC | PS1 *cnaA* seq F |
| JOHE54709/YES229 | TGGTGTATGGTGTCGAAACTGA | PS1 *cnaA* seq R |
| JOHE54440/YES192 | AGTTGATTGGACAAGCGCCT | PS1 *cnaB* seq F |
| JOHE54441/YES193 | GTGCTCTGTTGTAATGCGATGG | PS1 *cnaB* seq R |
| JOHE54442/YES194 | AGCACATGGTGGCTTGATTTC | PS1 *cnaC* seq F |
| JOHE54443/YES195 | CAAGCAGCATCCATGTCTTGG | PS1 *cnaC* seq R |
| JOHE54443/YES196 | TGTGCTCAACACAACATGTCG | PS1 *cnbR* seq F |
| JOHE54443/YES197 | AGCTTGCTGAAGACTCACTCC | PS1 *cnbR* seq R |
| JOHE53990/YES89 | GCCTACTTGAGCAAGCATCGT | PS3 *fkbA* seq F |
| JOHE53989/YES88 | ATGGGCTTGTTGCATCGC | PS3 *fkbA* seq R |
| JOHE56489/YES498 | GTCGTGTGGCACATTGCC | PS3 *cnaA* seq F |
| JOHE56490/YES499 | GGCGAATACGCATGCCGA | PS3 *cnaA* seq R |
| JOHE56491/YES500 | TGCTGTGGAAACCTCCACG | PS3 *cnaB* seq F |
| JOHE56492/YES501 | ATGTCGCAAGAGCGCCTTG | PS3 *cnaB* seq R |
| JOHE56493/YES502 | GAGAGGAGAGAAAAGGAGGGAA | PS3 *cnaC* seq F |
| JOHE56494/YES503 | CTGTATGCCCGCATCGTCA | PS3 *cnaC* seq R |
| JOHE56495/YES504 | CCGCTTGTGCTATCAGTGATTC | PS3 *cnbR* seq F |
| JOHE56496/YES505 | CCCATCCTGACAGACTTGG | PS3 *cnbR* seq R |
| JOHE53556/YES29 | AACCGTGAGAAGATGACCCA | PS3 *actA* probe F |
| JOHE53557/YES30 | TCCAAACGCAAGATAGCGTG | PS3 *actA* probe R |
| JOHE54916/YES253 | GAAGCGAATGGACGACGAAT | PS3 positive control probe F |
| JOHE54917/YES254 | AGAGCGTTAACTCTGTCCGT | PS3 positive control probe R |
| JOHE54857/YES231 | ACATGGCCTTTGATTTGCCT | PS3 5' *fkbA* probe F |
| JOHE54858/YES232 | AGGCCTATCATCATTCACTCTTGT | PS3 5' *fkbA* probe R |
| JOHE53457/YES18 | GAATCTCTCCTGGCGATGG | PS3 *fkbA* probe exon2 F |
| JOHE53459/YES20 | TCCCAACCCTTGATGACTTG | PS3 *fkbA* probe exon2 R |
| JOHE54914/YES251 | TTGTCTGTTGGTGAGAAGGC | PS3 *fkbA* probe exon3 F |
| JOHE54915/YES252 | GGGTAACCACGCTCACCATA | PS3 *fkbA* probe exon3 R |
| JOHE54859/YES233 | ACTAAACTCCTTGCCTGGACA | PS3 3' *fkbA* probe F |
| JOHE54860/YES234 | CAACCCTGCTACAGTAGATGC | PS3 3' *fkbA* probe R |
| JOHE56182/YES434 | GAACGCTTCTTGGCTGAGAG | PS3 negative control probe F |
| JOHE56183/YES435 | GTGGGCTGGATTGACTTCG | PS3 negative control probe R |
| JOHE56188/YES440 | GAGGCAGCCCTACTCCTATG | PS3 *fkbA* near gene probe F |
| JOHE56189/YES441 | ACGACGCTCATCGTTGGATA | PS3 *fkbA* near gene probe R |
| JOHE55830/YES365 | TGCCCATACCTAATGGCAGT | *PS3_001333* probe F |
| JOHE55831/YES366 | TTACTGCTGATGTGGCAGGT | *PS3_001333* probe R |
| JOHE55746/YES337 | GCCACACATCAACCCACATT | *PS3_001332* probe F |
| JOHE55747/YES338 | TCCAATGGAGGAGAGGAGGA | *PS3_001332* probe R |
| JOHE55750/YES341 | ACTGCCATCACTTGAAGCAC | *PS3_001331* probe F |
| JOHE55751/YES342 | TTGTGAACCTTGCTGGATGC | *PS3_001331* probe R |
| JOHE55752/YES343 | GCGACAGATCAAGCGTATCC | *PS3_001330* probe F |
| JOHE55753/YES344 | GGCCTGCATCTTCGTTCTTT | *PS3_001330* probe R |
| JOHE55752/YES343 | GCGACAGATCAAGCGTATCC | *PS3 patA* probe F |
| JOHE55753/YES344 | GGCCTGCATCTTCGTTCTTT | *PS3 patA* probe R |
| JOHE53992/YES91 | GCTGTGTCACGAAATGTGCA | PS6 *fkbA* seq F |
| JOHE53993/YES92 | ATGGGATTGCTGCATCGC | PS6 *fkbA* seq R |
| JOHE53973/YES72 | GGACTAGAAGACGCTCTCTCA | PS6 *cnaA* seq F |
| JOHE53974/YES73 | GAGCCTGAGCTTGAGCTC | PS6 *cnaA* seq R |
| JOHE53975/YES74 | GCCGACAACAAGCCATACTG | PS6 *cnaB* seq F |
| JOHE53976/YES75 | GTCTTCACGGTGGCCTTC | PS6 *cnaB* seq R |
| JOHE53977/YES76 | ATGCCAACATGTCTCACTCG | PS6 *cnaC* seq F |
| JOHE53978/YES77 | CCCAATAGTAGCCTGACACAC | PS6 *cnaC* seq R |
| JOHE53979/YES78 | CGTGTGCTTGTCAGGTTCAG | PS6 *cnbR* seq F |
| JOHE53980/YES79 | ATGATGCTGAAACTCACGCG | PS6 *cnbR* seq R |
| JOHE54407/YES168 | GGTGTTACCCACACTGTTCC | PS6 *actA* probe F |
| JOHE54408/YES169 | CCAGCCATATCCAAACGCAA | PS6 *actA* probe R |
| JOHE54869/YES243 | AATAGTCTTGTCTCATGCAGACT | PS6 *GremLINE1* probe F |
| JOHE54870/YES244 | GCACCAAGAGAGAGACGAG | PS6 *GremLINE1* probe R |
| JOHE54865/YES239 | AATGCACAAAGTCTCGGAGT | PS6 5' *fkbA* probe F |
| JOHE54866/YES240 | TCCCTTCTTCGATCATGTGTCA | PS6 5' *fkbA* probe R |
| JOHE54405/YES166 | GAATTGCTCCTGGTGATGGC | PS6 *fkbA* probe exon2 F |
| JOHE54406/YES167 | TCGAGAGTACCAACGTAGTGG | PS6 *fkbA* probe exon2 R |
| JOHE54924/YES261 | TCTGTTGGTGAGAAGGCCAA | PS6 *fkbA* probe exon3 F |
| JOHE54925/YES262 | GGGTAACCACGTTCACCATA | PS6 *fkbA* probe exon3 R |
| JOHE54867/YES241 | ACGTAAACCACCAAAGTCACC | PS6 3' *fkbA* probe F |
| JOHE54868/YES242 | AGCTCAGATGTTTATGGCTTGC | PS6 3' *fkbA* probe R |
| JOHE56260/YES442 | TCCGCAGAGGATCTTGAGAC | PS6 negative control probe F |
| JOHE56260/YES443 | AGTAGACGGCGGGATGATTT | PS6 negative control probe R |
| JOHE56180/YES432 | GCAAGTGATGCGATGTCTGA | PS6 *fkbA* near gene probe F |
| JOHE54869/YES243 | AATAGTCTTGTCTCATGCAGACT | PS6 *GremLINE1* probe F |
| JOHE54870/YES244 | GCACCAAGAGAGAGACGAG | PS6 *GremLINE1* probe R |
| JOHE56181/YES433 | CTCTTGCTGTGGGTTGGATG | PS6 *fkbA* near gene probe R |
| JOHE55756/YES347 | GCTGCTGATACATCCAGTGC | *PS6_003792* probe F |
| JOHE55757/YES348 | ATTGGAAGCAGCAGCTTGAG | *PS6_003792* probe R |
| JOHE55758/YES349 | GCTGCCATGTTCTACGTTGT | *PS6_003791* probe F |
| JOHE55759/YES350 | CGCCATCCAGGTAGTGTTTG | *PS6_003791* probe R |
| JOHE55760/YES351 | TTGCAGCCGAGATGCAGATA | *PS6_003790* probe F |
| JOHE55761/YES352 | TCCATGCTCGTTTGGAGATT | *PS6_003790* probe R |
| JOHE55762/YES353 | GATAGAGCGCCACCATCAAC | *PS6_003789* probe F |
| JOHE55763/YES354 | CTCTCAGGCTGAAACTGTGC | *PS6_003789* probe R |
| JOHE55764/YES355 | GACTACGATGGCTGGGACAT | *PS6_003786* probe F |
| JOHE55765/YES356 | CAGCTCGGCATAATGCTTCA | *PS6_003786* probe R |
| JOHE55766/YES357 | TGGATAACACTCGCCAGGAA | *PS6_003784* probe F |
| JOHE55767/YES358 | TCCTCTCTCTCTTGCTGCAC | *PS6_003784* probe R |
| JOHE55768/YES359 | ATTGAAGAAGCCCGCGACAT | *PS6_003783* probe F |
| JOHE55769/YES360 | CGACGAGAAGATGGTGGAGA | *PS6_003783* probe R |
