# Supplementary material for: Epimutations driven by RNAi or heterochromatin evoke transient antimicrobial drug resistance in pathogenic Mucor fungi
Source: PLoS Biol. 2026 Feb 2;24(2):e3003598. doi: 10.1371/journal.pbio.3003598 (PMC12863538; doi:10.1371/journal.pbio.3003598)
Supplement: S1 Raw Images — (DOCX) [file pbio.3003598.s022.docx]

**Supplementary Information**

**Epimutations driven by RNAi or heterochromatin evoke transient antimicrobial drug resistance in fungi**

Ye-Eun Son^1^, Carlos Pérez-Arques^1^, and Joseph Heitman^1*^

*^1^ Department of Molecular Genetics and Microbiology,* *Duke University Medical Center, Durham, North Carolina, United States of America*

**^*^**heitm001@duke.edu

**Uncropped scans of blots**

**FK506-resistant epimutants exhibit silencing of FKBP12 protein expression.**

**(A, B)** Western blot analysis of FKBP12 protein expression in *M. bainieri* (A) or *M. atramentarius* (B). PS3: E1–E9, epimutants; E1P4–E9P24, revertants. PS6: E1 and E2, epimutants; E1P32 and E2P28, revertants.
