# Supplementary material for: Epimutations driven by RNAi or heterochromatin evoke transient antimicrobial drug resistance in pathogenic Mucor fungi
Source: PLoS Biol. 2026 Feb 2;24(2):e3003598. doi: 10.1371/journal.pbio.3003598 (PMC12863538; doi:10.1371/journal.pbio.3003598)
Supplement: Attachment — Submitted filename: Response to Reviewers_final.docx [file pbio.3003598.s023.docx]

**Please note that the line numbers referenced in our response correspond to those in the clean version of the revised manuscript.**

-------------------------------------------------------------------------------------------------------------------------------

Reviewer #1:

The manuscript entitled "Epimutations driven by RNAi or heterochromatin evoke antifungal drug resistance in human fungal pathogens" explores epigenetic mechanisms underlying antifungal resistance in *Mucor* species, focusing on RNAi-dependent and heterochromatin-mediated silencing pathways. The topic is highly relevant to the field of antifungal therapy and microbial pathogenesis, particularly in the context of mechanisms of drug resistance. The work presents intriguing findings that contribute to our understanding of how fungi can adapt to antifungal pressure through heritable epimutations. However, several aspects of the manuscript require clarification and revision before it can be considered for publication. Below, I provide a detailed evaluation of the manuscript, including comments on the clarity, methodology, data interpretation, and the overall contribution to the scientific literature.

▷ We appreciate the insightful comments from this reviewer. We carefully checked the reviewer’s comments and revised the manuscript accordingly. We hope that we have addressed all concerns raised by this reviewer.

Use of the term "human fungal pathogens":

The manuscript repeatedly refers to the studied species and strains as "human fungal pathogens." However, the specific *Mucor* strains used (e.g., *Mucor bainieri* and *Mucor atramentarius*) are not recognized as clinically relevant pathogens, as there is no evidence that they have been isolated from human patients. Moreover, the virulence assays were conducted using severely immunocompromised mice, which may not accurately reflect pathogenic potential in typical human infections. Therefore, the use of the term "human fungal pathogens" in the title and throughout the text is misleading and should be revised. If the authors wish to make conclusions about mechanisms of antifungal resistance in human pathogens, they should validate their findings using strains from clinically relevant genera such as *Candida*, *Cryptococcus*, or *Aspergillus*, or specifically in pathogenic Mucorales like *Rhizopus*, *Lichtheimia*, or *Mucor* species with confirmed patient-derived origins.

▷ We appreciate the reviewer’s observation. Following this comment, we revised the term "human fungal pathogens” in both title and context.

**Line 1-2**: Epimutations driven by RNAi or heterochromatin evoke transient antimicrobial drug resistance in fungi.

**Line 41-44**: These findings reveal that antifungal resistance arising through distinct, transient epimutation pathways involving RNAi or heterochromatin, highlighting adaptive AMR strategies employed by ubiquitous eukaryotic microbes.

**Line 479-483**: Notably, this mechanism operates in the non-pathogenic species M. bainieri and M. atramentarius species, a species with pathogenic potential, suggesting that heterochromatin-mediated resistance is conserved across diverse fungal taxa, spanning both unicellular yeasts and multinuclear hyphal species.

Interpretation regarding DNA methylation:

Lines 157-161. The authors conclude that DNA methylation is not involved in FK506 resistance based on the absence of canonical 5-methylcytosine DNA methyltransferases in the *M. bainieri* genome. First, was this also determined in *M. atramentarius*? This section reads somewhat like a "data not shown" statement, leaving unclear what specific experiments or analyses were performed to support the claim. It would be helpful if the authors could clarify the methods used (such as BLASTp searches or other approaches) and provide the relevant data or tables. While the prior study cited offers a thorough analysis of histone and DNA methyltransferases in Mucorales, it does not include genomic evidence from these particular species. Including this information would strengthen the manuscript and improve transparency. The lack of identifiable homologs to known methyltransferases is not sufficient to rule out the presence or functional relevance of DNA methylation. At a minimum, the claim should be softened to acknowledge that low or atypical forms of DNA methylation may still exist and could play a role in gene regulation, even in the apparent absence of canonical DNMTs.

▷ We are grateful for the reviewer’s insightful comment. **Based on this comment, we analyzed 5-methylcytosine (5mC) DNA methyltransferases in both** M. bainieri **and** M. atramentarius **and confirmed that no homologs are present (S3A Fig). In addition, we examined 5mC DNA methylation in wild-type strains of** M. bainieri **and** M. atramentarius **based on Nanopore sequencing data and found no detectable methylation signals (S3B Fig). This analysis has been incorporated in the revised version.**

**Line 169-176**: To determine which epigenetic mechanisms are involved in FK506 resistance in *M. bainieri*, we first evaluated the potential role of 5mC DNA methylation. Whole-genome sequencing data revealed the absence of canonical DNA 5-methylcytosine methyltransferases (DNMT1, DIM-2, RID-1, DNMT4, and DNMT5), consistent with a previous study (**S3A Fig**) [30]. Furthermore, 5mC analysis via Nanopore sequencing in the two *Mucor* wild types detected no cytosine methylation, while a clear 5mC signal was detected in a *Cryptococcus neoformans* clinical isolate with the same analysis pipeline [31], suggesting that this mechanism is not involved in FK506 resistance (**S3B Fig**).

**Line 258-261**: **In *M. atramentarius*, canonical DNA 5-methylcytosine methyltransferases (DNMT1, DIM-2, RID-1, DNMT4, and DNMT5) were also absent, consistent with a previous study (S3A Fig) [29]. Moreover, 5mC analysis in the wild type detected no cytosine methylation, suggesting that DNA methylation is not involved in FK506 resistance (S3B Fig).**

**[Figure S3]**

Clarity on resistant isolation and frequency:

The manuscript lacks clarity on the scale and rigor of the screening efforts to isolate FK506 resistant isolates across different species. It is unclear how many spores or colonies were actually screened per species, and whether the observed resistant isolates in *M. bainieri* and *M. atramentarius* reflect a true biological prevalence or simply greater sampling effort. Especially in *M. atramentarius*, the sample size of potential H3K9 resistant isolates appears rather low (n = 1), which challenges the reproducibility and generalizability of the results. Furthermore, the absence of detected resistant isolates in other species raises questions about the generality of this mechanism in other fungal pathogens. Do these species possess gene silencing and chromatin modification proteins?

▷ Thank you for pointing this out. For each species tested, approximately 10⁵ spores were inoculated onto more than ten solid YPD plates and incubated for 1–2 weeks. From these cultures, resistant isolates were recovered by selecting hyphal sectors that continued to grow on YPD supplemented with FK506. In *M. bainieri*, nine out of ten independent resistant isolates exhibited RNAi- or heterochromatin-associated resistance, and in *M. atramentarius*, two out of ten isolates displayed heterochromatin-associated resistance. Although relatively rare, this phenotype was observed in both species.

With respect to the other *Mucor* species in which resistant isolates were not recovered, we repeated the screening at least three independent times. However, we acknowledge that the total number of colonies screened was smaller than in previously published large-scale epimutant screens and therefore cannot exclude the possibility that resistant isolates may occur at low frequency but were not detected due to sampling limitations. Additionally, several species (e.g., PS4, PS5, PS9, PS11, PS13) display intrinsic FK506 resistance, and PS12 grows poorly on solid media, which may further reduce the likelihood of recovering FK506-resistant epimutants under our assay conditions. As a result, we cannot conclusively determine whether the absence of resistant isolates reflects true biological differences or methodological constraints. Future comparative genomic and functional analyses across the broader *Mucor circinelloides* species complex will be required to clarify whether heterochromatin-mediated resistance is conserved or varies among species.

**Line 500-503**: To isolate FK506-/rapamycin-resistant strains, 10^5^ spores were inoculated onto more than ten solid YPD plate, supplemented with antifungal drug 1 μg/mL of FK506 (Astellas Pharma, Chuo, Tokyo, Japan) and 100 ng/ml of rapamycin (API Chem, Hangzhou, Zhejiang, Chian) and incubated for 3, 5, 7 days [15].

**Line 130-137**: In contrast, analysis of ten FK506-resistant isolates from *M.* *bainieri* revealed only one isolate with a nonsense mutation in *fkbA*, while the remaining nine exhibited no detectable sequence changes in *fkbA*, or the genes encoding calcineurin A or B subunits, suggesting a possible epigenetic regulation (**Table 1**). Similarly, in *M. atramentarius*, among ten FK506-resistant isolates, three harbored nonsense mutations in *fkbA*, one contained a synonymous mutation, two had splice-site point mutations, one had a start-loss mutation, and one had a point mutation in the calcineurin B subunit (*cnbR*) (**Table 1**).

Clarification and documentation of reference genomes:

The manuscript states that the genomes of *M. bainieri* CBS293.63 and *M. atramentarius* CBS202.28 were used as reference genomes in the sRNAs and ChIP sequencing subsections. However, it is unclear whether these genomes have been previously published, as no references could be found to support their prior availability. If these reference genomes were generated as part of the present study, the authors must include a dedicated subsection in the Materials and Methods detailing the procedures used. This should include information on DNA extraction, sequencing platforms and technologies used, assembly and annotation strategies and software, as well as quality control metrics for each step (e.g., read quality, assembly statistics, completeness assessment, repeat content). Proper documentation of genome generation is essential for reproducibility and to support any downstream analyses presented in the manuscript.

▷ We are grateful for the reviewer’s insightful comment. To address this point and further improve the manuscript, we generated high-quality reference genomes of *M. bainieri* CBS293.63 and *M. atramentarius* CBS202.28 using Oxford Nanopore sequencing. These new assemblies were used for mapping of sRNA and H3K9me2 ChIP-seq data. And, we also described relevant information in the Materials and Methods detailing the procedures, including DNA extraction, library preparation, sequencing platform and parameters, assembly and annotation pipelines. This additional information ensures reproducibility and provides transparency for the analyses presented in the manuscript.

**Line 509-519**: **Illumina sequencing and genome assembly**

*Mucor* mycelium were grown on YPD medium, harvested, frozen in liquid nitrogen, and ground into a find powder. Genomic DNA samples were prepared using Norgen Biotek Yeast/Fungi Genomic DNA Isolation Kit (Ontario, Canada). Genomic DNA libraries were constructed with Roche KAPA HyperPrep Kit. 150-bp paired-end reads were obtained utilizing the NovaSeq X Plus sequencing system (Illumina) at the Duke Sequencing and Genomic Technologies Core (SGT). Raw reads were checked for quality metrics using FastQC v0.11.7 and processed by fastp v0.23.4 to remove adapters and low-quality bases or reads. Genomes were assembled using SPAdes v3.15.5 set to isolate default mode. Genome assemblies were cleaned, sorted, masked, and annotated using the Funannotate v1.8.15 [63].

**Line 521-551**: **Nanopore sequencing and genome assembly**

A total of 10⁶ spores from *M. bainieri* and *M. atramentarius* wild-type strains were inoculated into liquid YPD medium and incubated overnight at 25 °C. Cells were harvested by centrifugation, washed with distilled water, frozen with liquid nitrogen, and ground with a mortar and pestle. High-molecular-weight (HMW) DNA was extracted from each sample using the Monarch HMW DNA Extraction Kit for Tissue (NEB, Ipswich, MA, USA) according to the manufacturer’s protocol. DNA concentration and quality were assessed using a NanoDrop spectrophotometer (ThermoFisher, Waltham, MA, USA) and a Qubit fluorometer with the Qubit dsDNA High Sensitivity kit (ThermoFisher). DNA integrity was confirmed by CHEF electrophoresis performed at 6.0 V in a 1% agarose gel for 18 hours, which revealed intact HMW DNA. Libraries were prepared using the ligation sequencing kit V14 (SQK-LSK114; Oxford Nanopore Technologies, Oxford, United Kingdom) and sequenced on MinION R10.4.1 flow cells (ONT; Oxford Nanopore Technologies) following to the manufacturer’s protocol in-house in our laboratory.

POD5 base calling was performed with the Dorado basecaller (v0.5.2; Oxford Nanopore Technologies, https://github.com/nanoporetech/dorado). Demultiplexing and BAM-to-FASTQ conversion were carried out using Dorado demux (v0.5.2). The resulting FASTQ files were error-corrected with Canu (v2.2) and assembled using Flye (v2.9.5-b1801) [64, 65]. The expected genome size was set to 50 Mb, and a minimum overlap length of 5 kb was required between reads. The assembled genome was subsequently polished in three iterations using Pilon (v1.24; Broad Institute, https://github.com/broadinstitute/pilon/releases/tag/v1.24) with default parameters. Repetitive sequences were identified using RepeatMasker (v4.1.5; https://www.repeatmasker.org) with default parameters and genome annotation was performed using Funannotate (v1.8.17) with the masked genome assembly and strain-specific RNA-seq alignments [63]. This pipeline produced high-quality genome assemblies, with *M. bainieri* assembled into 30 contigs (N50 = 6 Mb; 98.7% BUSCO completeness; 9,781 predicted genes) and *M. atramentarius* into 33 contigs (N50 = 2 Mb; 98.8% BUSCO completeness; 11,597 predicted genes).

ChIP-seq positive controls (Figure 4 and 5)

Regarding methodology, how were the positive controls for H3K9me2 ChIP selected? Are these transposons? A brief description of these elements and criteria for their selection would be informative. As stated above, including an additional repeat content track in the genome visualization would help assess these concerns and contextualize the sRNA and heterochromatin patterns.

▷ We really appreciate this valuable comment. For the positive controls in H3K9me2 ChIP experiments, we relied on previously characterized transposable elements, specifically GremLINE, which had been identified in *M. lusitanicus* (Navarro-Mendoza et al., Current Biology 2019), further characterized in *Mucor* species (Pérez-Arques et al., PLoS Genetics 2020), and later examined in additional detail (Navarro-Mendoza et al., PNAS 2024). In PS6, we were able to locate GremLINE elements and therefore used these regions as positive controls. By contrast, in the PS3 assemblies, GremLINE elements could not be detected. In this case, we instead utilized our sRNA-seq data to identify three candidate regions with strong small RNA enrichment and subsequently validated these by ChIP-qPCR. The regions that displayed both sRNA accumulation and H3K9me2 enrichment were then used as positive controls. In line with the reviewer’s suggestion, we have now incorporated genome and transposable element (TE) tracks into the genome browser visualization and provided a more detailed description of the selection criteria in the revised manuscript.

**Line 631-644**: For H3K9me2 ChIP-qPCR, GremLINE1 transposon elements were used as positive controls in *M. atramentarius* [30]. However, our analysis of the *M. bainieri* genome suggests the GremLINE1 elements are absent in the Illumina-based genome assembly. Instead, to develop a positive control for the H3K9me2 ChIP-qPCR analysis in *M. bainieri*, we hypothesized that the loci enriched for H3K9m2 should at least partially overlap with the genomic regions enriched for small RNAs. Specifically, we first identified three genomic loci enriched for small RNAs based on our sRNA analysis and then designed primers targeting these loci and tested them for the presence of H3K9me2 signals using ChIP-qPCR. One of the three loc, *PS3_010814* (identified in Illumina-based genome assembly deposited under NCBI SRA PRJNA1277536), exhibited consistent H3K9me2 enrichment across samples including WT, epimutants, and revertants, and was selected as the positive control for the subsequent H3K9me2 ChIP-qPCR analyses. The *actA* gene, which lacks both small RNA accumulation and heterochromatin marks, was used as a negative control.

**[Figure 4]**

**[Figure 5]**

Interpretation of *in vivo* results:

The evidence presented by the authors does not exclude alternative explanations such as de novo resistance, which is observed in some wild-type and revertant colonies. More importantly, animals were sacrificed only four days post-infection, which is considerably shorter than the average time required for reversion in vitro, which is around 30 days? in some cases. It is not clear what exactly constitutes a "passage" in this context: are these taken from a continuous culture every day (single overnight subcultures), a full growth cycle, or something else? The methods section does not explicitly define this term or the passage protocol.

This limited infection timeframe may complicate efforts to determine whether resistance stability in the host is greater or lesser than that observed in vitro, which appears to be the intended focus of this experiment. The authors should consider tempering their conclusions or extending the duration of in vivo infection.

▷ We really appreciate this valuable comment. Based on this comment, we performed additional analyses to address these concerns. First, whole-genome sequencing of FK506-resistant isolates recovered from each organ of WT-, epimutant-, and revertant-infected mice confirmed that resistance was not due to Mendelian mutations (**S3-S6 Table**). Second, to address the concern regarding the short passage timeframe, we extended our *in vivo* passage experiments to include longer durations (2 weeks with or without low-dose cyclophosphamide, and 4 weeks without cyclophosphamide). Resistant isolates were recovered under all tested conditions, and their stability was further evaluated by ChIP-qPCR for H3K9me2 enrichment. These additional results, now presented in **Figures 6** and **7**, provide a more comprehensive view of resistance *in vivo* and clarify the temporal stability of epimutants during infection.

**Line 313-316**: Sequencing of the *fkbA* locus in FK506-resistant isolates recovered from each organ of WT-, epimutant-, and revertant-infected mice revealed no mutations, with sequences identical to the wild type, indicating that these isolates represent epimutants rather than Mendelian mutants (**S9B Fig** and **S3 Table)**.

**Line 325-342**:	To further examine whether epimutant stability persists over longer periods of infection, we extended our *in vivo* assays by administering a lower dose of cyclophosphamide (an immunosuppressant) or without cyclophosphamide treatment and then assessed FK506 resistance and heterochromatin-associated stability after two or four weeks. Unlike the results observed at 4 days post-infection, fungal burden at 2 weeks post-infection with lower or no cyclophosphamide, and at 4 weeks post-infection without cyclophosphamide, was detected only in the liver and spleen, but not in the brain or lung (**S11A-S11C Figs**). Next, when fungal colonies recovered from the liver and spleen were tested on FK506-containing medium, resistance was retained in colonies derived from epimutant-infected mice at frequencies greater than 50% under all conditions tested (**Figs 7A, 7C, and 7E**). With resistant colonies from liver and spleen, we sequenced the *fkbA* locus, confirming that there were no mutations, with sequences identical to the wild type (**S11D-S11F Figs** and **S4-S6 Tables**). Furthermore, ChIP-qPCR analysis revealed that FK506-resistant epimutants exhibited H3K9me2 enrichment at the *fkbA* locus (**Figs 7B, 7D,** and **7F**). Together, these results demonstrate that heterochromatin-mediated antifungal resistance in *M. atramentarius* is stably maintained during prolonged host infection, independent of genetic mutations, and is instead sustained by mitotically inherited H3K9me2 marks.

We have also revised the Methods section to clearly define “passage” as described.

**Line 505-507**: These were then serially subcultured overnight for several rounds, after which the passaged cells were spotted onto YPD or YPD supplemented with FK506 to confirm phenotypic plasticity.

Minor comments

- The manuscript lacks sufficient detail on data analysis methodology. For every major analysis step, detailed documentation of software versions, parameters, and any custom scripts is important. Providing this information (whether in the main text, materials and methods, or figure legends) is essential to enable reproducibility, facilitate comparison with standard workflows and help reviewers and readers assess the reliability of the results.

▷ Thank you for pointing this out. Based on this comment, we have now described the detailed protocol for nanopore sequencing and genome assembly, sRNA, and ChIP-sequencing in the Materials and methods section.

**Line 509-519**: **Illumina sequencing and genome assembly**

*Mucor* mycelium were grown on YPD medium, harvested, frozen in liquid nitrogen, and ground into a find powder. Genomic DNA samples were prepared using Norgen Biotek Yeast/Fungi Genomic DNA Isolation Kit (Ontario, Canada). Genomic DNA libraries were constructed with Roche KAPA HyperPrep Kit. 150-bp paired-end reads were obtained utilizing the NovaSeq X Plus sequencing system (Illumina) at the Duke Sequencing and Genomic Technologies Core (SGT). Raw reads were checked for quality metrics using FastQC v0.11.7 and processed by fastp v0.23.4 to remove adapters and low-quality bases or reads. Genomes were assembled using SPAdes v3.15.5 set to isolate default mode. Genome assemblies were cleaned, sorted, masked, and annotated using the Funannotate v1.8.15 [63].

**Line 521-551**: **Nanopore sequencing and genome assembly**

A total of 10⁶ spores from *M. bainieri* and *M. atramentarius* wild-type strains were inoculated into liquid YPD medium and incubated overnight at 25 °C. Cells were harvested by centrifugation, washed with distilled water, frozen with liquid nitrogen, and ground with a mortar and pestle. High-molecular-weight (HMW) DNA was extracted from each sample using the Monarch HMW DNA Extraction Kit for Tissue (NEB, Ipswich, MA, USA) according to the manufacturer’s protocol. DNA concentration and quality were assessed using a NanoDrop spectrophotometer (ThermoFisher, Waltham, MA, USA) and a Qubit fluorometer with the Qubit dsDNA High Sensitivity kit (ThermoFisher). DNA integrity was confirmed by CHEF electrophoresis performed at 6.0 V in a 1% agarose gel for 18 hours, which revealed intact HMW DNA. Libraries were prepared using the ligation sequencing kit V14 (SQK-LSK114; Oxford Nanopore Technologies, Oxford, United Kingdom) and sequenced on MinION R10.4.1 flow cells (ONT; Oxford Nanopore Technologies) following to the manufacturer’s protocol in-house in our laboratory.

POD5 base calling was performed with the Dorado basecaller (v0.5.2; Oxford Nanopore Technologies, https://github.com/nanoporetech/dorado). Demultiplexing and BAM-to-FASTQ conversion were carried out using Dorado demux (v0.5.2). The resulting FASTQ files were error-corrected with Canu (v2.2) and assembled using Flye (v2.9.5-b1801) [64, 65]. The expected genome size was set to 50 Mb, and a minimum overlap length of 5 kb was required between reads. The assembled genome was subsequently polished in three iterations using Pilon (v1.24; Broad Institute, https://github.com/broadinstitute/pilon/releases/tag/v1.24) with default parameters. Repetitive sequences were identified using RepeatMasker (v4.1.5; https://www.repeatmasker.org) with default parameters and genome annotation was performed using Funannotate (v1.8.17) with the masked genome assembly and strain-specific RNA-seq alignments [63]. This pipeline produced high-quality genome assemblies, with *M. bainieri* assembled into 30 contigs (N50 = 6 Mb; 98.7% BUSCO completeness; 9,781 predicted genes) and *M. atramentarius* into 33 contigs (N50 = 2 Mb; 98.8% BUSCO completeness; 11,597 predicted genes).

**Line 554-564**: Approximately 1–5 mg of harvested cells was suspended in 300 μL Tissue & Cell Lysis Buffer (Lucigen, Middleton, WI, USA) and 1 μL proteinase K (ThermoFisher). The mixture was incubated at 65°C for 45 min, followed by the addition of 150 μL MPC Protein Precipitation Reagent (Lucigen). After centrifugation, the pellet was washed with 70% ethanol and resuspended in distilled water, and genomic DNA (gDNA) quality was analyzed using a Qubit and a NanoDrop (ThermoFisher). The promoter, open reading frame, and terminator regions of the *fkbA* gene were amplified by polymerase chain reaction (PCR) using primers listed in **S2 Table**. The purified PCR products were sequenced using Long read sequencing with ONT at Plasmidsaurus Inc. (Eugene, CA, USA) and analyzed against the reference *fkbA* sequence using Clustal Omega (v1.2.3).

**Line 585-588**: ~. Filtered reads were then mapped to the reference genomes (*M. bainieri* CBS293.63; PRJNA1277536 or *M. atramentarius* CBS202.28; PRJNA1277544) using BBMap with default parameters (last modified September 15, 2022) [67].

**Line 590-594**: The data was subsequently visualized in the Integrative Genome Viewer (IGV v2.16.0), where the Y-axis represents the average read coverage per 20-bp window [68]. Peak calling was performed with MACS3 using the --nomodel option with an extension size of 147 bp.

- Figure 3 and 5. Are the sRNAs exclusively spreading toward FKBP12, or is there evidence of broader targeting? Expanding the genomic window or providing supplementary views of upstream regions is recommended, as the data show a prominent H3K9me2 peak and sRNA signal immediately adjacent on the left edge of the displayed window, which appears abruptly truncated.

▷ We are grateful for the reviewer’s insightful comment. In response, we re-mapped the sRNA and H3K9me2 data onto the newly assembled genome generated from Nanopore sequencing. Using this updated assembly, we expanded the genomic window to include upstream regions adjacent to the *fkbA* locus.

**[Figure 3A]**

**[Figure 5A]**

- Abstract. Line 34: why strikingly? In vivo stability was assayed at 4 days post-infection, which is considerable less time than most resistant isolates take to revert in vitro. It would be striking if they were not stable under the conditions assayed.

▷ Thank you for raising this concern. We have revised the Abstract to remove this wording.

**Line 40-41**: Heterochromatin-mediated *fkbA* epimutants exhibited stability during *in vivo* infection, suggesting epimutation could impact pathogenesis.

- Introduction. Lines 50-55: The authors describe well-known genetic mechanisms of antifungal resistance in *Candida*, *Aspergillus*, and *Cryptococcus* species, but omit examples from the Mucorales. Given that the manuscript focuses entirely on these species, a brief mention of known classical resistance mechanisms within Mucorales would improve cohesion and set the stage more clearly for the current study.

▷ We really appreciate this valuable comment. Based on this comment, we added examples from the Mucorales.

**Line 57-62**: Genetic changes include point mutations (missense/nonsense) and insertions and deletions (indels) in drug target genes or drug transporter genes, or their regulators, as well as large-scale chromosomal alterations such as copy number variation and aneuploidy. These changes confer resistance in several human fungal pathogens including Candida albicans, Cryptococcus neoformans, Aspergillus fumigatus, and Mucor circinelloides [10-13].

- Introduction. Line 52. The authors list ERG11 and CYP51 as if they represent distinct antifungal targets, but these are homologous genes encoding the same protein (sterol C14-demethylase) targeted by azoles.

▷ We are grateful to the reviewer for pointing this out. Following this comment, we have revised the text and removed the redundant gene list to avoid confusion.

**Line 58**: ~ in drug target genes or drug transporter genes, or their regulators, ~

- Introduction. Line 78: The statement that "acquired antifungal resistance in *Mucor* spp. continue to undermine treatment efficacy" is not supported by clinical evidence. Most Mucorales exhibit intrinsic resistance to certain antifungals but reports of acquired resistance during treatment are exceedingly rare.

▷ We are grateful for the reviewer’s insightful comment. Based on this comment, we revised this text.

**Line 84-87**: Given both the severity and widespread incidence of mucormycosis, a deeper understanding of intrinsic resistance and the potential for acquired antifungal resistance is urgently needed to guide proper and efficient therapeutic interventions.

- Methods. Line 458: Authors should clarify if these are technical or biological triplicates.

▷ Thank you for pointing it out. Based on this comment, we revised it.

**Line 630-631**: qPCR was performed in technical triplicate with primers described in **S2 Table**.

-------------------------------------------------------------------------------------------------------------------------------
Reviewer #2:

Fungal infections present an emerging threat and resistance to antifungals is a significant barrier to developing new treatments. In this manuscript, Son and Heitman describe how Mucorales leverages small RNAs and heterochromatin to establish resistance to FK506 and Rapamycin. This work builds on previous studies from the Heitman lab demonstrating how Mucorales circinelloides (Navarro-Mendoza et al.,PNAS,2024) uncouples RNAi from H3K9 methylation to silence LINE elements. Viewed in this context, the results that this manuscript showcase are interesting and distinctive from their previous studies. I really like the comparative analysis of adaptation to FK506 by searching across the clade. It is remarkable interesting that there is sufficient diversity within the Mucorales clade that there are situations where RNAi and H3K9me can be coupled (or uncoupled) to establish silencing.

The authors clearly show that epimutants do arise when surveying drug-resistant isolates in different Mucorales species. By passaging individual colonies in the absence of FK506 for extended periods of time the authors demonstrate reversion of the phenotype, drug resistance, a classic sign of epimutation. Surprisingly, some of these isolates have small RNAs that target *fkbA*, others have only H3K9me over the same locus, and still a third category of fungal adapters exhibit configurations of adaptation where both pathways are coupled. It is unclear how this response was initiated but the phenomenology is clearly documented in this manuscript.

The manuscript is well-written; the data is for the most part quite clear in cataloguing these widespread adaptive phenomena of anti-fungal resistance. I also liked the *in vivo* approach to determining whether anti-fungal resistance remains stable in mice. This was overall a nice touch to the paper demonstrating the clinical relevance of antifungals. Despite my generally positive impression of the paper, I had some comments that I hope the authors will engage with:

▷ We are grateful for the reviewer’s constructive feedback. We carefully considered the comments and revised the manuscript accordingly. We hope that the revisions address the points raised.

CONCEPTUAL ISSUES:

1. Have the authors surveyed LINE elements (GremLINES) across the different species described in the current manuscript to see if the data they observe in the case of *Mucor circinelloides* also applies to the other species? I am specifically referring to the uncoupling between H3K9me and RNAi. Since the authors already have P0 datasets, I assume this would not involve any additional experiments but only further analysis. My expectation would be that *M.banieri* does not show this uncoupling between H3K9me and RNAi as a first approximation based on their epimutation data.

▷ We are grateful for the reviewer’s insightful comment. Based on your suggestion, when we examined GremLINE elements across species, we found that GremLINE loci are present in M. atramentarius, similar to M. lusitanicus and M. circinelloides, but are absent from the M. bainieri genome. In all three species that harbor these elements, GremLINEs are located within or adjacent to the predicted centromeric regions, consistent with their established association with pericentromeric heterochromatin in previous studies. While *M. atramentarius* exhibits strong H3K9me2 enrichment at GremLINEs, the precise relationship between RNAi and heterochromatin at these loci may differ and will require further investigation to clarify potential species-specific variation.

In contrast, *M. bainieri* lacks GremLINE elements entirely. Instead, we predicted putative centromeric regions based on the degenerate CEN motif reported in *M. lusitanicus* (Navarro-Mendoza et al., Curr Biol., 2019) together with H3K9me2 ChIP-seq enrichment and found that these regions are enriched for a distinct repeat, TE00000158, which shows strong H3K9me2 enrichment. This pattern is markedly different from the other species, suggesting that *M. bainieri* may represent a species-specific centromeric repeat landscape and RNAi–heterochromatin relationship that differs from the GremLINE-associated uncoupling observed in *M. lusitanicus*.

**Line 460-474:** Beyond FK506-induced dynamics at the *fkbA* locus, our comparative genomic analyses across four Mucor species provide additional insight into how RNAi and heterochromatin interplay may differ among species. GremLINE elements are present in *M. atramentarius*, *M. lusitanicus*, and *M. circinelloides*, where they reside within or near putative centromeric regions, but are absent from the *M. bainieri* genome (**S14 Fig**). The centromeric GremLINEs in these species consistently show strong H3K9me2 enrichment, supporting their association with heterochromatic domains. Notably, *M. bainieri* exhibits a distinct architecture. Although it lacks GremLINEs, its putative centromeric regions are enriched for a different repeat, TE00000158, which is associated with strong H3K9me2. This pattern diverges sharply from the GremLINE-associated uncoupling observed in *M. circinelloides*, raising the possibility that *M. bainieri* may have evolved a species-specific centromeric repeat landscape and RNAi-heterochromatin relationship. Collectively, these comparative observations suggest that the interplay between RNAi and heterochromatin may not be conserved and may vary across species, likely influenced by species-specific differences in repeat landscapes and centromere architecture.

**[Figure S14]**

2. The authors also in their PNAS manuscript, Navarro-Mendoza et al. 2024 perform a phylogenetic analysis to propose that coupling is absent because of the lack of a critical H3K9me-RNAi subunit - Chp1. It seems like the authors are setup to do comparative analysis between genomes, which they could apply to the present study. The authors must provide some ideas for why these different *Mucor* species behave so differently? Are there differences in epigenetic pathways between species that might explain this observation?

▷ We really appreciate this valuable comment. Based on this, we conducted comparative analysis of RNA interference, H3K9 methylation-based heterochromatin formation, and RNA-induced transcriptional silencing (RITS) components across four *Mucor* species (*M. lusitanicus*, *M. circinelloides*, *M. bainieri*, and *M. atramentarius*) in **S10 Fig** and address this in the revised Discussion section.

**Line 405-474:** The interplay between RNAi and histone methylation raises key questions about the sequence and causality of these silencing events. Preexisting heterochromatin can initiate RNAi, as seen in centromeres and transposable elements in S. pombe and Arabidopsis thaliana, where transcripts from heterochromatic loci are processed into siRNAs that reinforce silencing [59]*.* In S. pombe, small RNAs bound to Argonaute form the RNA-induced transcriptional silencing (RITS) complex with the chromodomain protein Chp1 and the bridging protein Tas3. Chaperone proteins such as Hsp90 stabilize RITS complex, which recruits Clr4 to nascent transcripts, thereby facilitating heterochromatin formation [59, 60]. Stc1 associates with Ago1 and recruits the chromatin-modifying CLRC complex (Clr4-Rik1-Cul4-Dos1-Dos2), underscoring its critical role in connecting RNAi with heterochromatin formation [61]. Meanwhile, euchromatic loci may sporadically generate double-stranded RNA, initiating RNAi and leading to heterochromatin deposition via a positive feedback loop involving the RITS complex and Clr4 [62]. [62]. Interestingly, in our study, we identified FK506-resistant epimutants with varying combinations of siRNA accumulation and H3K9me2 enrichment at the *fkbA* locus (**Fig 4**). These findings support several non-exclusive models: (1) RNAi-driven initiation of heterochromatin, (2) heterochromatin-facilitated RNAi, or (3) other mechanisms.

To explore the first hypothesis, we conducted a comparative analysis of RNAi, H3K9 methylation-based heterochromatin formation, and RITS components across M. bainieri, M. atramentarius, M. lusitanicus, and M. circinelloides to assess the potential coupling between RNAi and heterochromatin pathway. As reported previously in M. lusitanicus [30], the RITS components Chp1 and Tas3 are absent across all four *Mucor* species, whereas the bridging protein Stc1, the histone methyltransferase Clr4, and CLRC complex subunits (Rik1, Cul4, and Raf1) are conserved (**S12 Fig**). Thus, although elements of the coupling machinery such as Stc1 and CLRC are conserved, the apparent absence of a complete RITS complex suggests that the connection between RNAi and heterochromatin may operate in a partial or context-dependent manner. Under FK506 selective pressure, RNAi may be initiated at the *fkbA* locus, leading in some cases to the establishment of heterochromatin, while in others, RNAi may act independently. Even when heterochromatin is formed, weak or transient interactions between RNAi and heterochromatin could result in heterochromatin-only epimutants.

Second, preexisting heterochromatin could facilitate RNAi, serving as a template for small RNA generation. To explore this possibility, we analyzed the distribution of H3K9 dimethylation and sRNA in wild-type strains of four *Mucor* species (*M. bainieri*, *M. atramentarius*, *M. lusitanicus*, and *M. circinelloides*). As shown in **S13 Fig**, discrete H3K9me2 islands were detected downstream of *fkbA* in all four species, suggesting that preexisting heterochromatin on transposon-rich regions may spread toward the *fkbA* locus under FK506 selective pressure and recruit the RNAi machinery to generate small RNAs. Similar phenomena have been reported in other organisms. In *S. pombe*, for example, caffeine stress triggers redistribution of H3K9 methylation and evokes sRNA generation [17]. Interestingly, these H3K9me2 islands lacked detectable sRNAs in *M. lusitanicus* and *M. circinelloides*, whereas the co-occurrence of H3K9me2 and sRNAs was observed in *M. bainieri* and *M. atramentarius*, raising the possibility that the relationship between RNAi and heterochromatin formation may interact differently across species.

Third, RNAi and heterochromatin formation may occur simultaneously under FK506 pressure but act independently without functional coupling. A previous study reported that RNAi and heterochromatin formation can function independently to repress GremLINE1 transposable elements and maintain genome stability in *M. lusitanicus* [30]. Thus, it is possible that, upon FK506 exposure, these machineries were independently activated at the same genomic region, with RNAi generating sRNAs at the *fkbA* locus and heterochromatin spreading from adjacent transposon-rich regions, resulting in their apparent coexistence without direct mechanistic interaction. Taken together, future studies analyzing pathway-specific mutants will be critical to determine the directionality and mechanistic coupling or uncoupling of these epigenetic processes.

Beyond FK506-induced dynamics at the *fkbA* locus, our comparative genomic analyses across four *Mucor* species provide additional insight into how RNAi and heterochromatin interplay may differ among species. GremLINE elements are present in *M. atramentarius*, *M. lusitanicus*, and *M. circinelloides*, where they reside within or near putative centromeric regions, but are absent from the *M. bainieri* genome (**S14 Fig**). The centromeric GremLINEs in these species consistently show strong H3K9me2 enrichment, supporting their association with heterochromatic domains. Notably, *M. bainieri* exhibits a distinct architecture. Although it lacks GremLINEs, its putative centromeric regions are enriched for a different repeat, TE00000158, which is associated with strong H3K9me2. This pattern diverges sharply from the GremLINE-associated uncoupling observed in *M. circinelloides*, raising the possibility that *M. bainieri* may have evolved a species-specific centromeric repeat landscape and RNAi-heterochromatin relationship. Collectively, these comparative observations suggest that the interplay between RNAi and heterochromatin may not be conserved and may vary across species, likely influenced by species-specific differences in repeat landscapes and centromere architecture.

**[Figure S12]**

**[Figure S13]**

3. If the genome sequences are too similar, the authors could compare transcriptomes to give some sense of why exposure to FK506+Rapamycin elicits different epimutational response across species. These experiments are important since they are foundational to the point of this manuscript which otherwise for the most part only confirm what the Heitman lab has very elegantly shown is a pervasive feature of Mucorales genomes.

▷ We are grateful for the reviewer’s insightful comment. We agree that comparative transcriptomic analyses would provide valuable mechanistic insights into species-specific differences in epimutational responses to FK506+Rapamycin. While these experiments were not included in the present study, we fully recognize their importance and plan to pursue them in future studies. In this manuscript, our primary objective was to establish and validate the occurrence and stability of heterochromatin-associated resistance in multiple Mucorales species, thereby extending and complementing previous findings.

4. Line 174 - While indeed sRNAs between 21-24 nt are observed, it does seem that there is a distinct distribution of sRNAs at the *fkbA* genes relative to the surrounding genes. At *fkbA*, the population of sRNAs is predominantly 21 and 23 nt, while at neighboring genes there are more 23+24 nt sRNAs. This appears to be true of the 2 epimutants in *M.atramentarius* as well. Do the authors have an explanation for this? Might it point to an underlying mechanism of epimutation establishment?

▷ We are grateful for the reviewer’s insightful comment. We observed distinct sRNA size distributions between the *fkbA* locus and its neighboring genes. While the precise mechanistic basis remains unclear, size-specific biases in sRNA populations may arise from multiple mechanisms. We have now expanded the Discussion section to address these possibilities in more detail.

**Line 384-404**: Our data revealed that the size distribution of sRNAs at the *fkbA* locus differs from those mapped to neighboring genes (**Fig 3, S5** and **S7 Figs**). The predominant sRNAs derived from *fkbA* were 21 and 24 nt, whereas most neighboring genes displayed stronger accumulation at 23–24 nt. Previous work in *Mucor* proposed that different sRNA size classes may serve distinct roles in silencing, with 21-nt sRNAs acting as the predominant species involved in target mRNA degradation [52]. In this context, the increased abundance of 21 nt sRNAs derived specifically from *fkbA* suggests that FK506-resistant epimutants may preferentially generate the sRNA class most directly linked to efficient target degradation. A similar phenomenon was also reported at the *pyrG* and *pyrF* loci, where sRNAs fell within the 21–24 nt range but showed locus differences. *pyrG* sRNAs were enriched at 21 and 24 nt, whereas *pyrF* sRNAs were predominantly 23–24 nt [16]. Such variation may result from multiple, non-mutually exclusive mechanisms. First, differential usage of Dicer paralogs may contribute. In *M. circinelloides*, DCL2 produces 23–24 nt siRNAs, whereas DCL1 can result in more heterogeneous size distributions including 21 nt [53, 54]. Similarly, in the model plant *Arabidopsis thaliana*, DCL4 primarily generates 21-nt siRNAs, while DCL3 is responsible for 24-nt siRNAs involved in transcriptional silencing [55, 56]. Second, substrate properties of the transcripts, including secondary structure, GC content, and overall architecture, may influence Dicer processing efficiency and cleavage-site preference, thereby generating distinct size profiles [57, 58]. These observations suggest that even within the canonical RNAi framework, locus-specific features may shape distinct sRNA size distributions.

5. The authors suggest that reversion in cases where RNAi and H3K9me is coupled is because of a fitness cost related to ATP utilization. I find this model to be very underwhelming. There are many things that cells do which are pretty energy intensive and I would argue that a single epimutation at the fkbA locus is unlikely to be the primary driver of fitness defects. There are many simpler mechanisms that explain the differences in the stability of the epigenetic state but the cellular energy-based hypothesis is particulsarly unfounded.

▷ Thank you for pointing this out. In response to this comment, we removed this part in the revised discussion section.

ANALYSIS ISSUES:

1. Line 242 – This is a qualitative statement, which is not itself an inherent problem, but as currently presented it is hard for the reader to assess this statement using the figures. Figure 3A and/or 4C would need to be referenced to substantiate this claim, but as the scale of the y-axes of these genome browser plots differ greatly between the *M. bainieri* and *M. atramentarius* plots (0-500 vs 0-20), it is difficult to directly compare. The authors should at minimum include an additional genome browser plot which allows for direct comparison of sRNAs at the fkbA locus between the two species. Furthermore, comparison of the plots in figures S3 and S4 suggest that there are many more sRNAs being produced in *fkbA* flanking genes in *M. bainieri* than in *M. atramentarius*. Difference in sRNA abundance between the species could be a relevant and interesting observation, and the authors should comment on this difference.

▷ We are grateful for the reviewer’s insightful comment. Based on this comment, we have revised the genome browser plots to compare directly the y-axis scales across species. We also now highlight in the text that *M. bainieri* shows a higher accumulation of sRNAs at flanking genes compared to *M. atramentarius*.

**Line 270-273**: Interestingly, while the spreading pattern resembled that observed in heterochromatin-associated epimutants of *M. bainieri* (**Figs 3A** and **5A**), *M. bainieri* accumulated markedly higher levels of sRNAs at genes flanking the *fkbA* locus compared to *M. atramentarius* (**S4** and **S6 Figs**).

**[Figure 3A]**

**[Figure 5A]**

2. Generally, more quantification in the text will make the paper more easily readable. For example, in 3B or 5B highlighting fold changes in small RNAs would help readers understand what makes this phenomenology interesting. It would also help readers compare data where the authors find three different bins of adaptation. Are there any correlations between lower levels of small RNAs and the appearance of H3K9me? Lower transcription would lead to reduced small RNA production as well (qualitatively this does not seem to be the case looking at the data but nevertheless these quantifications could guide potential hypotheses).

▷ We are grateful for the reviewer’s insightful comment. Following this suggestion, we have added quantitative descriptions of sRNA abundance at representative loci in the revised Results section. However, our analysis did not support a simple correlation between sRNA levels and H3K9me2 enrichment. While loci with strong H3K9me2 accumulation generally exhibited transcriptional repression, the corresponding levels of sRNAs varied widely among genes. This suggests that the relationship between RNAi activity and heterochromatin establishment is not strictly proportional but may depend on locus-specific factors such as chromatin context or accessibility.

**Line 180-183**: In epimutants E5–E9, *fkbA*-specific sRNAs strongly accumulated to ~1,000 RPM, whereas in WT and most revertants no reads were detected, with only a minimal signal (~1 RPM) observed in E9 revertants (**Fig 3B**).

**Line 193-194**: For example, at *PS3_001333*, sRNAs reached ~2,595 RPM in epimutants E1-E3 but was largely absent in WT (0.15 RPM) and in revertants (1–1.6 RPM).

L**ine 217-222**: H3K9me2 levels at the *fkbA* locus were markedly increased in epimutants E1–E4, with fold enrichment ranging from ~5-fold to over 100-fold relative to WT, although the magnitude varied across loci and strains. By contrast, H3K9me2 enrichment in E8 and its revertant E8P28 was negligible and comparable to WT. These results indicate that histone modification is associated with unstable antifungal drug resistance in some epimutants (E1-E4) and not others.

**Line 276-277**: ~ with fold enrichment ranging from ~12-fold to over 60-fold relative to WT (**Fig 5B**).

3. Colors of small RNAs and H3K9me keep changing across figures. It would make the paper much more readable to have consistent formatting. Specifically, I am referring to figures 3 and 4.

▷ Thank you for pointing this out. Based on this helpful suggestion, we have revised the figures to use constant colors for small RNAs and H3K9me across all relevant panels (including Figures 3-5).

**[Figure 3A]**

**[Figure 5A]**

-------------------------------------------------------------------------------------------------------------------------------
Reviewer #3:

This manuscript examines how different species in the *Mucor circinelloides* complex develop resistance to the antifungal drug FK506, focusing on the roles of RNAi and heterochromatin (H3K9 methylation) in gene silencing. The authors extend previous findings from *M. lusitanicus* and *M. circinelloides* to three additional species—*M. janssenii*, *M. bainieri*, and *M. atramentarius*—and compare both Mendelian (genetic) and non-Mendelian (epigenetic) resistance mechanisms. The study is well structured and addresses an important question in antifungal resistance.

A key and well-supported finding is that different *Mucor* species rely on distinct mechanisms to acquire FK506 resistance:

1. In *M. janssenii*, all 10 resistant isolates carry mutations in *fkbA*, with no evidence of epimutational silencing.
2. In *M. bainieri*, 9 of 10 isolates are epimutants that show reversible resistance and no mutations in *fkbA*. These fall into three mechanistic groups: RNAi-only, H3K9me2-only, or both.
3. In *M. atramentarius*, 8 isolates are Mendelian mutants, and 2 are candidate epimutants showing H3K9 methylation at *fkbA*, consistent with heterochromatin-based silencing.

These results clearly demonstrate species-specific preferences for either mutation-driven or epigenetic resistance and show that epimutational silencing itself can be achieved through different combinations of regulatory mechanisms. The study is technically strong and well executed. The combination of sequencing, small RNA profiling, and ChIP analysis is appropriate and convincing. The classification of epimutants into mechanistic subtypes is clearly presented. The use of reversion assays further supports the epigenetic nature of the observed resistance.

However, despite the quality of the data, I am not fully convinced that the findings are novel enough for publication in PLOS Biology. The role of RNAi in antifungal resistance in *Mucor* has already been reported, and the presence of H3K9 methylation-based heterochromatin is established. Moreover, the cooperation between RNAi and heterochromatin is a known phenomenon in other systems such as *Schizosaccharomyces pombe* and *Arabidopsis thaliana*. Demonstrating this cooperation in *Mucor* is interesting, but not entirely unexpected.

What could make this study more impactful is the observation that some species rely on RNAi alone, while others require cooperation between RNAi and heterochromatin. This raises an important and unresolved question: Why do some *Mucor* species or strains rely solely on RNAi, while others require both RNAi and heterochromatin? This question touches on fundamental differences in how gene silencing pathways are coordinated. One possibility is the presence or absence of species-specific bridging factors that connect the RNAi machinery with the histone methylation system. In *S. pombe*, for example, Stc1 links Argonaute-siRNA complexes to the Clr4 methyltransferase. A similar cofactor might exist in some *Mucor* species but not in others.

An alternative hypothesis, however, is that these observed differences between species are not due to fundamentally distinct silencing mechanisms, but rather to differences in frequency. For example, RNAi + heterochromatin-based silencing may exist at low frequency in all *Mucor* species but was simply not detected due to the limited number of epimutants analyzed. If true, this would suggest that all MCC species are capable of using the same epigenetic tools, but to varying degrees. It would therefore be important to ask: Do the authors have the statistical power to confidently exclude this frequency-based explanation?

▷ We are thankful for the reviewer’s thoughtful comments. Based on this comment, we performed a comparative analysis of RNAi, H3K9 methylation-based heterochromatin formation, and RNA-induced transcriptional silencing (RITS) components across four *Mucor* species (*M. lusitanicus*, *M. circinelloides*, *M. bainieri*, and *M. atramentarius*) (**S10 Fig**). This analysis confirmed that all four species share most of the core components required for RNAi and H3K9me2 establishment, including Stc1, the putative bridging factor that in *S. pombe* connects Ago1–siRNA complexes to the Clr4 methyltransferase. However, the canonical RITS components Chp1 and Tas3 were absent from all four species, consistent with previous reports. We further address this point in the revised Discussion section, where we outline several additional hypotheses concerning the interplay between RNAi and histone methylation.

**Line 422-435:** To explore the first hypothesis, we conducted a comparative analysis of RNAi, H3K9 methylation-based heterochromatin formation, and RITS components across M. bainieri, M. atramentarius, M. lusitanicus, and M. circinelloides to assess the potential coupling between RNAi and heterochromatin pathway. As reported previously in M. lusitanicus [30], the RITS components Chp1 and Tas3 are absent across all four *Mucor* species, whereas the bridging protein Stc1, the histone methyltransferase Clr4, and CLRC complex subunits (Rik1, Cul4, and Raf1) are conserved (**S12 Fig**). Thus, although elements of the coupling machinery such as Stc1 and CLRC are conserved, the apparent absence of a complete RITS complex suggests that the connection between RNAi and heterochromatin may operate in a partial or context-dependent manner. Under FK506 selective pressure, RNAi may be initiated at the *fkbA* locus, leading in some cases to the establishment of heterochromatin, while in others, RNAi may act independently. Even when heterochromatin is formed, weak or transient interactions between RNAi and heterochromatin could result in heterochromatin-only epimutants.

We next examined what underlying features might account for these interspecies differences. When we examined GremLINE elements,LINE-type transposable elements known to be enriched near centromeres, were compared across species (Nvarro-Mendoza et al., Curr Biol., 2019). We found that GremLINE loci are present in *M. atramentarius*, similar to *M. lusitanicus* and *M. circinelloides*, but are absent from the *M. bainieri* genome. In all three species containing these elements, GremLINEs are located within or adjacent to the predicted centromeric regions. While *M. atramentarius* exhibits strong H3K9me2 enrichment at GremLINEs, the precise relationship between RNAi and heterochromatin at these loci may differ and will require further investigation to clarify potential species-specific variation.

In contrast, *M. bainieri* lacks GremLINE elements entirely. Instead, we predicted putative centromeric regions based on a degenerate version of the CEN motif reported in *M. lusitanicus* (Navarro-Mendoza et al., Curr Biol., 2019) together with H3K9me2 ChIP-seq enrichment and found that these regions are enriched for a distinct repeat, TE00000158, which shows strong H3K9me2 enrichment accompanied by abundant sRNA production. This pattern is markedly different from the other species, suggesting that *M. bainieri* may represent a species-specific centromeric repeat landscape and RNAi–heterochromatin relationship that differs from the GremLINE-associated uncoupling observed in *M. lusitanicus*. We further discuss these possibilities in the revised Discussion section.

**Line 460-474:** Beyond FK506-induced dynamics at the *fkbA* locus, our comparative genomic analyses across four *Mucor* species provide additional insight into how RNAi and heterochromatin interplay may differ among species. GremLINE elements are present in *M. atramentarius*, *M. lusitanicus*, and *M. circinelloides*, where they reside within or near putative centromeric regions, but are absent from the *M. bainieri* genome (**S14 Fig**). The centromeric GremLINEs in these species consistently show strong H3K9me2 enrichment, supporting their association with heterochromatic domains. Notably, *M. bainieri* exhibits a distinct architecture. Although it lacks GremLINEs, its putative centromeric regions are enriched for a different repeat, TE00000158, which is associated with strong H3K9me2. This pattern diverges sharply from the GremLINE-associated uncoupling observed in *M. circinelloides*, raising the possibility that *M. bainieri* may have evolved a species-specific centromeric repeat landscape and RNAi-heterochromatin relationship. Collectively, these comparative observations suggest that the interplay between RNAi and heterochromatin may not be conserved and may vary across species, likely influenced by species-specific differences in repeat landscapes and centromere architecture.

**[Figure S13]**
