# Supplementary material for: Epimutations driven by RNAi or heterochromatin evoke transient antimicrobial drug resistance in pathogenic Mucor fungi
Source: PLoS Biol. 2026 Feb 2;24(2):e3003598. doi: 10.1371/journal.pbio.3003598 (PMC12863538; doi:10.1371/journal.pbio.3003598)
Supplement: Attachment — Submitted filename: Final_Response to Reviewers_121925.docx [file pbio.3003598.s025.docx]

**Please note that the line numbers referenced in our response correspond to those in the clean version of the revised manuscript.**

-------------------------------------------------------------------------------------------------------------------------------

Reviewer #1:

I have carefully evaluated the revised manuscript and the detailed point-by-point responses provided by the authors. I am satisfied that all major concerns raised in the previous review round have been thoroughly and adequately addressed. The authors have significantly strengthened the manuscript by providing new genomic and methylation analyses, clarifying resistant isolate screening, and generating fully documented high-quality reference genomes that support all downstream sequencing work. They also improved the rigor of the ChIP and sRNA analyses, extended and validated the in vivo experiments to rule out genetic resistance, and enhanced overall methodological transparency and contextual framing. Collectively, these revisions fully address the concerns raised and considerably improve the clarity and scientific robustness of the study.

▷ We sincerely thank the reviewer for the thorough evaluation of the revised manuscript and for the positive and encouraging comments. We are pleased that the revisions adequately addressed all major concerns and improved the clarity and scientific rigor of the study. We have carefully corrected all minor typographical errors noted by the reviewer in the revised manuscript.

Just a few minor comments about typos found during the new reading:

"find powder" → should be "fine powder" Occurs in the Illumina sequencing methods description.

"ground into a find powder" appears twice

First in Illumina prep, again in Nanopore prep—both should be "fine powder."

▷ Thank you for pointing it out. Based on this comment, we revised it.

**Line 511**: ~ and ground into a fine powder.

"Chian" → should be "China" In the description of rapamycin source ("Zhejiang, Chian").

▷ Thank you for the guidance. Based on this comment, we revised it.

**Line 502-503**: ~ (API Chem, Hangzhou, Zhejiang, China) ~

Sentence fragment or missing word in "One of the three loc,"

"loc" is an incomplete word; likely intended as "loci."

▷ Thank you for noting this. Based on this comment, we revised it.

**Line 639**: One of the three loci, ~

Inconsistent gene name formatting ("H3K9m2" vs. "H3K9me2")

At least one instance uses "H3K9m2," missing the "e."

▷ Thank you for pointing it out. Based on this comment, we revised it.

**Line 636**: H3K9me2 should at least partially overlap ~

"following to the manufacturer's protocol" → should be "according to the manufacturer's protocol"

▷ Thank you for the guidance. Based on this comment, we revised it.

**Line 534**: ~ according to the manufacturer’s protocol in our laboratory.

Grammatically incorrect phrasing in Nanopore sequencing workflow.

"These were then serially subcultured overnight for several rounds"

Awkward and ambiguous wording; unclear subject and duration.

▷ Thank you for pointing it out. Based on this comment, we revised it.

**Line 505**: These were then serially subcultured overnight for multiple consecutive rounds,~

-------------------------------------------------------------------------------------------------------------------------------

Reviewer #2:

I really appreciate the authors detailed responses to my questions. In particular, I want to highlight the tremendous value that I thought the analysis of RNAi/CLRC/RITS subunits added to strengthen the manuscript and also the comparative genomic analysis of GremLINES. It is very interesting that these elements are missing in *M.bainieri*. These analyses clearly highlight the power of comparative genomic analysis, reveal important differences across *Mucor* species and at least provide opportunities to envision possibilities for why RNAi and H3K9me show such wide mechanistic disparities during adaptation. The only concern I have is that the experiments are clearly an opening for comparative transcriptomic analysis. The authors state pursuing transcriptomic analysis to identify adaptation mechanisms in future studies. Upon reading the reviewer responses, I am still left thinking that the transcriptomic data would be a welcome addition to the current manuscript. This is my only concern because it is also an opportunity to set this manuscript apart from earlier work and really use the power of comparative analysis across species. That being said, I am still a fan of the paper. The additional experiments, the extended discussion are all welcome additions that make the paper a very engaging read.

▷ We sincerely thank the reviewer for the thoughtful, detailed, and encouraging comments, and for highlighting the value of the comparative genomic analyses presented in this study. We appreciate the reviewer’s interest in the potential of comparative transcriptomic analyses to further investigate adaptation mechanisms. While we agree that such analyses would be valuable and could further strengthen the study, we plan to pursue them in future studies. We thank the reviewer again for the positive assessment and for the constructive suggestions that helped improve the clarity and impact of the manuscript.

-------------------------------------------------------------------------------------------------------------------------------

Reviewer #3:

The revised version contains further analysis and discussion that address my comments convincingly.

▷ We sincerely thank the reviewer for the positive and encouraging comments.
